# Complement in Tumourigenesis and the Response to Cancer Therapy

**DOI:** 10.3390/cancers13061209

**Published:** 2021-03-10

**Authors:** Rebecca M. O’Brien, Aoife Cannon, John V. Reynolds, Joanne Lysaght, Niamh Lynam-Lennon

**Affiliations:** 1 Department of Surgery, Trinity St. James’s Cancer Institute, Trinity Translational Medicine Institute, Trinity College Dublin and St. James’s Hospital, Dublin 8, Ireland; obrier20@tcd.ie (R.M.O.); cannona@tcd.ie (A.C.); reynoljv@tcd.ie (J.V.R.); jlysaght@tcd.ie (J.L.); 2 Cancer Immunology and Immunotherapy Group, Trinity St. James’s Cancer Institute, Trinity Translational Medicine Institute, Trinity College Dublin and St. James’s Hospital, Dublin 8, Ireland

**Keywords:** complement, cancer, cancer treatment, therapeutic response

## Abstract

**Simple Summary:**

Increasing evidence supports a role for complement in the development of cancer and the response to cancer treatments. Dysregulated complement expression within the tumour microenvironment has been linked to the suppression of anti-tumour immunity and poor clinical outcomes. Complement signals have been demonstrated to alter the immune milieu, promote proliferation and facilitate metastasis. Targeting complement signalling in combination with current treatments may have the potential to achieve improved control of tumour growth.

**Abstract:**

In recent years, our knowledge of the complement system beyond innate immunity has progressed significantly. A modern understanding is that the complement system has a multifaceted role in malignancy, impacting carcinogenesis, the acquisition of a metastatic phenotype and response to therapies. The ability of local immune cells to produce and respond to complement components has provided valuable insights into their regulation, and the subsequent remodeling of the tumour microenvironment. These novel discoveries have advanced our understanding of the immunosuppressive mechanisms supporting tumour growth and uncovered potential therapeutic targets. This review discusses the current understanding of complement in cancer, outlining both direct and immune cell-mediated roles. The role of complement in response to therapies such as chemotherapy, radiation and immunotherapy is also presented. While complement activities are largely context and cancer type-dependent, it is evident that promising therapeutic avenues have been identified, in particular in combination therapies.

## 1. Introduction

A dynamic relationship exists between the immune system and cancer, owing to the fact that a system designed to defend the host and maintain homeostasis has the potential to promote and foster malignant transformation [1,2]. Complement, an innate inflammatory system, is no exception to this paradox [3]. Traditionally, the elimination of foreign antigens was considered the primary, if not sole function of complement, however we now understand that complement activities extend beyond this [4]. The complement system, for instance, plays an important role coordinating adaptive immune responses, as an opsonin, in synapse elimination and during angiogenesis [5,6,7,8]. Several studies have demonstrated that complement is also capable of recognising and eliminating malignant cells [9]. The net effect of these diverse functions renders the complement system a key player in immune surveillance and homeostasis [4]. The delicate equilibrium between developing tumours and the immune system is well documented, with evasion of immune destruction defined as a hallmark of cancer [10]. In line with reports of an altered immune milieu in several human cancers, dysregulation of the complement system in the cancer setting has been observed [11,12,13,14,15]. More recently, pro-oncogenic roles for complement cascade components have been described [16,17]. Analysis of the current literature suggests that the complement system has a dual role in malignancy [4,18] and whether complement protects against or enables tumour pathogenesis may depend on the context of the tumour microenvironment (TME) [19]. This review will discuss the current understanding of the roles played by complement components in cancer, in particular focusing on how they may influence response to cancer therapy.

## 2. The Complement System

In 1901, Jules Bordet described complement as a heat-labile factor that augmented antibody-mediated bacterial lysis [20]. Subsequent discoveries have since established that complement is not a single entity but represents a family of many proteins [21]. The complement system is composed of approximately 50 soluble and membrane-bound complement effectors, regulators and receptors, with the main complement proteins numbered C1-9 [4]. Many complement precursors exist as zymogens, which require cleavage in order to gain functionality [22]. The C3 and C5 convertase enzymes are central to the complement cascade, cleaving C3 and C5 respectively to generate anaphylatoxins (C3a, C5a) and opsonins (C3b, C5b) [4,23]. The small anaphylatoxin molecules are potent inflammatory mediators with many effector functions [22,24]. Complement proteins are primarily produced by the liver before systemic dissemination via the bloodstream, however, we now understand that T cells, macrophages, endothelial cells and more recently, cancer cells, are capable of complement production.

### 2.1. Complement Activation Pathways

**Figure 1:**
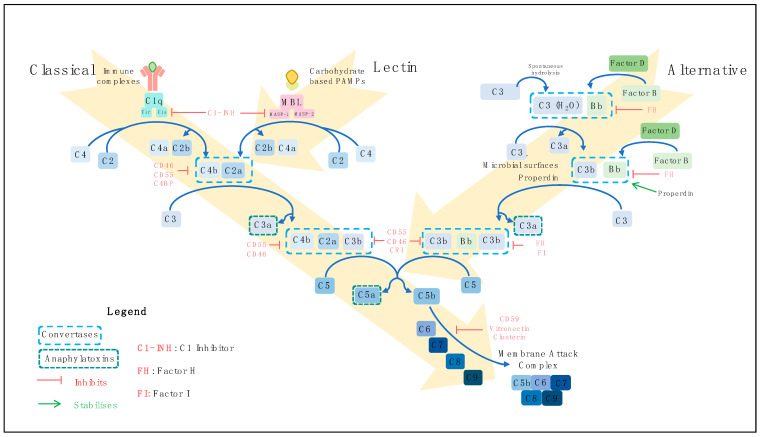
Complement activation pathways. There are three routes by which the complement system can become activated: the classical, the lectin and the alternative pathways. Classical pathway activation is initiated primarily by antigen-antibody immune complexes. C1q of the C1 complex (C1q, C1r and C1s) interacts with the fragment crystallistion (Fc) portion of antigen-bound immunoglobulins, activating C1r, which subsequently cleaves and activates C1s. Activated C1s cleaves C4 into C4a and C4b, and C2 into C2a and C2b leading to assembly of C4bC2a, the C3 convertase. Carbohydrate-based pathogen-associated molecular patterns (PAMPs) trigger activation of the lectin pathway. Mannose-binding lectin (MBL), ficolins or collectins recognise PAMPs, activating MBL-associated serine proteases (MASPs). Similar to the classical pathway, C4 and C2 are cleaved to generate C4bC2a. The classical and lectin complement activation pathways converge at this point to cleave C3 into the potent anaphylatoxin C3a, and C3b, which joins the C3 convertase to form C4bC2aC3b, the C5 convertase. Cleavage of C5 yields the C5a anaphylatoxin and C5b, which polymerises with C6, C7, C8 and C9 to form the membrane-attack complex (MAC). This inserts into target cell membranes to induce lysis. Spontaneous hydrolysis of C3 into C3H_2_O occurs in the alternative pathway. Cleavage of factor B (FB) by factor D yields Bb, which associates with C3H_2_O to form a C3 convertase. Cleavage of C3 and FB produces C3b and Bb, respectively. The binding of properdin to microbial surfaces recruits C3b, facilitating the assembly of the C3 convertase (C3bBb), and initiating pathway activation. Subsequent cleavage of C3 produces C3b, which combines with the C3 convertase to form a C5 convertase (C3bBbC3b). From this point, the terminal pathway is initiated to assemble the MAC, similarly to the classical and lectin pathways. Complement activation is regulated at various stages of the pathways by several membrane-bound complement regulatory proteins (Complement receptor 1 (CR1), CD46, CD55 and CD59) and circulating factors (C1-inhibitor (C1-INH), factor H (FH), factor I (FI), C4-binding protein (C4BP), clusterin and vitronectin), which are depicted in red, and properdin, which stabilises the alternative pathway C3 convertase.

There are three pathways by which the complement system may be activated, the classical, the lectin and the alternative pathways (Figure 1). The classical pathway is principally initiated when C1q of the C1 complex (C1q, C1r and C1s) recognises antigen-antibody (Immunoglobulin (Ig) G or IgM containing) immune complexes, but several antibody-independent signals such as C-reactive protein and viral proteins can activate this pathway also [25,26,27,28,29,30,31]. Viral and bacterial carbohydrate-based pathogen-associated molecular patterns (PAMPs) activate the lectin pathway by binding to mannose-binding lectin (MBL), ficolins or collectins [32,33,34,35]. In the alternative pathway, C3 is spontaneously hydrolysed to C3H_2_O in a process known as ‘tick-over’ [36,37]. Bacterial and yeast polysaccharides and damaged tissue are among the initiators of this pathway [38,39]. The binding of properdin to target microbial surfaces can facilitate the assembly of the alternative pathway C3 convertase [40,41]. The alternative pathway also acts as an amplification loop for the classical and lectin pathways [23,42]. Irrespective of the course of activation, the three activation pathways converge to initiate the terminal pathway. This pathway culminates with the assembly of complement components C5b–C9 to form a membrane attack complex (MAC) [43,44]. MAC insertion into target cell membranes can trigger lysis known as complement-dependent cytotoxicity (CDC) [45], or at sublytic doses may activate signalling pathways to promote cell survival [46,47].

### 2.2. Regulation of Complement Activation

Activation and amplification of the complement system induces a powerful inflammatory response, necessitating a regulatory system to avoid damage to host cells. This is achieved by a number of soluble and membrane-bound effector molecules which modulate various critical stages of the pathway, including the widely expressed membrane-bound complement regulatory proteins (mCRPs) (Table 1) and the fluid phase proteins C1 inhibitor, C4b-binding protein, factor H (FH) and factor I (FI) [35,48,49]. The alternative pathway C3 convertase is stabilised when bound by properdin [50].

### 2.3. Functions of Complement

MAC-induced lysis is the central cytotoxic event resulting from complement cascade activation, however, complement opsonins and anaphylatoxins also contribute to host defence. C3a and C5a exert their biological functions by binding to their respective receptors, the C3a receptor (C3aR) and the C5a receptor 1 (C5aR1/CD88), two G protein-coupled receptors [64,65]. A second, lesser understood C5aR, C5aR2 (previously C5L2) also exists [66]. In contrast, this 7-transmembrane receptor is uncoupled to G-proteins but is capable of recruiting β-arrestins [67,68,69]. C3a and C5a have been demonstrated to induce chemotaxis of mast cells [70,71] and eosinophils [72], with C5a also acting as a chemoattractant for macrophages [73], monocytes [74] neutrophils [74,75], basophils [76] and T and B lymphocytes [77,78]. Complement opsonins such as the C3 fragments C3b and iC3b aid phagocytosis by allowing recruited phagocytes to adhere to target cells via complement receptor 1 (CR1), complement receptor 3 (CR3), complement receptor 4 (CR4) and complement receptor Ig [4,79]. The phagocytic response to immune complexes may be enhanced by C5a-mediated upregulation of activating fragment crystallisation (Fc) γ receptors (FcγR) on the surface of phagocytes [24,80].

Complement cascade components also play key roles in orchestrating adaptive immunity. The complement receptors CR1 and CR2 are essential in the generation of B cell and follicular dendritic cell responses [81]. B cell responses are augmented by the binding of antigen opsonized by C3d to CR2, which leads to enhanced signalling through the B cell receptor and subsequent lowering of the threshold for activation [82,83,84]. In addition, complement components play roles in the priming and differentiation of T cells and provide survival signals to naïve T cells in an autocrine fashion [85,86,87,88]. Complement-mediated regulation of immune cells has been demonstrated within the TME [16,89]. The implication of these interactions on the efficacy of cancer treatment will be discussed later in this review.

## 3. The Complement System and Cancer

### 3.1. Activation of the Complement System in Cancer

As a key mediator in host defence, traditionally complement has been considered a component of anti-tumour immune defence. Indeed, several studies have demonstrated that the system is activated systemically and within the TME. Increased MBL/MASP activity and MBL levels have been observed in the serum of patients with colorectal cancer when compared to non-cancer controls [90]. In lung cancer cell lines, incubation with normal human serum has been demonstrated to activate complement and give rise to increased C5 deposits when compared to cell lines derived from the normal bronchial epithelium [91]. Furthermore, increased C5a levels have been reported in the plasma of non-small cell lung cancer (NSCLC) patients when compared to healthy controls, suggesting that local activation of complement is followed by systemic diffusion [91]. Complement components are deposited in many tumour tissues, for instance C4d, a C4-derived fragment, has been reported in oropharyngeal squamous cell carcinomas [92] and follicular and mucosal-associated lymphoid tissue lymphomas [93]. Similarly, others have demonstrated that C3c is abundantly deposited in tumour tissue from glioblastoma multiforme patients, when compared to non-malignant controls [94]. In addition, this study reported deposition of the C5b–C9 MAC on tumour cells. The presence of MAC complexes in tumour tissue has also been reported in breast [95], gastric [96] and thyroid [97] cancers, and within ovarian cancer-associated ascitic fluid [98] demonstrating local complement activation up to advanced stages. The complex relationship between cancer cells and the MAC has recently been reviewed in detail, including the pro-lytic signals responsible for mediating the necrotic cell death induced by MACs [99].

Collectively, these observations provide evidence for tumour-induced activation of the complement system. Given the role of complement in immune defence, it may be expected that complement activation within the TME would associate with positive impact. Paradoxically, in some cancer types, complement system activity is observed to correlate with poor prognosis, including cervical [100], colorectal [101] and ovarian cancer [102]. These studies suggest that dysregulation of the complement system occurs in a number of human cancers. It is widely accepted that a chronic inflammatory state facilitates neoplastic transformation [1] and current evidence suggests that complement activity, even if primarily initiated as a mechanism of host defence, may become tumour promoting as a result of sustained inflammation within the TME.

### 3.2. Tumour Expression of Complement Regulators

In the process of tumour development, a range of mechanism exist to avoid immune destruction [10]. Host cells express mCRPs to limit complement activation and avoid damage to healthy tissue [49]. This strategy is exploited by cancer cells, which often express complement regulators at levels higher than those observed in non-malignant tissue [103,104,105,106]. For example, head and neck squamous cell carcinoma (HNSCC) cells express significantly elevated levels of CD46, CD55 and CD59, when compared to benign keratinocyte cells. Furthermore, tumour infiltrating lymphocytes (TILs) from HNSCC patients have significantly increased expression of mCRPs, when compared to those from healthy controls [13]. Although there is evidence for complement activation in cancer, the expression of mCRPs within the TME provides evidence for tumour evasion of complement activities. Importantly, this attempt by tumour cells to avoid complement-mediated lysis appears successful. Expression of mCRPs correlates with poor clinical outcomes in cancer more often than not [107]. An extensive overview of the contribution of mCRPs to tumour growth and their current status as biomarkers, is presented in a recent review by Geller and Yan [108].

Soluble complement regulators are also employed by tumours in a bid to regulate complement activation. Lung cancer cells produce and secrete FH, and FI is secreted by NSCLC cells providing them with protection from complement-mediated lysis [109,110,111]. Together with evidence for the expression of mCRPs in the TME, the expression of fluid-phase complement effectors by cancer cells is consistent with an active attempt by tumours to evade detection by complement. A recent study has demonstrated that mice deficient in FH spontaneously develop hepatic tumours [112]. Furthermore, analysis of The Cancer Genome Atlas (TCGA) demonstrated that increased expression of FH was associated with improved patient disease-free survival (DFS) and overall survival (OS) when compared to patients expressing unaltered levels of FH, while mutations in FH were associated with poor OS [112]. These data illustrate that altered expression of complement regulators contributes to tumorigenesis and may have a prognostic impact.

### 3.3. Complement in Tumour Growth

Although the expression of complement regulators may allow tumour cells to proliferate ‘unchecked’ [108], a pan-cancer contribution of complement in directly enhancing tumour growth is yet to be, and is unlikely to be elucidated, largely due to the heterogeneity among cancer types and patients. Despite this, in recent years advances have been made in understanding the relationship between complement and cancer with several studies demonstrating pro-tumorigenic roles for complement. Several excellent reviews have recently discussed the role of complement in tumour growth and dissemination in detail [3,19,113].

#### 3.3.1. Murine Studies

The complement system was often overlooked as an integral component of the TME. A seminal paper in 2008 from Markiewski and colleagues however, provided the first clear evidence for complement in tumourigenesis. They demonstrated using a syngeneic TC-1 mouse model, that C3^−/−^, C4^−/−^ and C5aR^−/−^ mice have significantly reduced tumour growth [16]. It is well established that local cells within the TME, and the mediators they produce, play key roles in tumour growth [114]. In this model, they demonstrated that in the absence of C5a signalling, fewer myeloid-derived suppressor cells (MDSCs) migrated to tumours, allowing for increased infiltration of CD8^+^ T cells [16]. Since then, several others have demonstrated pro-tumour roles for complement, in particular providing evidence for complement modulation of the anti-tumour immune response (Table 2). Signalling through the C3a/C3aR and C5a/C5aR axes has been observed to remodel the TME, altering immune infiltrates and inducing immunosuppressive phenotypes. Complement-mediated regulation of T cell function is the most well described relationship between the complement system and an immune cell. This is likely due to the roles played by autocrine complement in T cell homeostasis, differentiation and metabolism [85,115,116,117].

Other key mechanisms and pathways distinct from the immune system have also been described. In a mouse model of ovarian cancer, activation of the C3aR and C5aR by their respective anaphylatoxins promotes proliferation of cancer cells by signalling through the PI3K/AKT pathway [118]. Enhanced proliferation and migration of melanoma and cutaneous squamous cell carcinoma (SCC) cells has also been ascribed to complement signalling [119,120,121,122]. Furthermore, complement has also been reported to induce angiogenesis [15,119,123] and foster acquisition of an epithelial-mesenchymal transition (EMT) phenotype by promoting expression of stemness genes [122], enhancing invasion [124,125,126] and increasing motility [127,128,129,130] of cells in several cancer types. Detailed overviews of the roles played by complement components in facilitating metastasis have recently been published by Ajona et al. [113] and Kochanek et al. [131].

It is notable that whilst the majority of murine studies have demonstrated pro-oncogenic roles for complement, a small number of studies have reported anti-tumour functions for complement. In mouse models of breast cancer C3, C5a, C1q and Factor P have been demonstrated to protect against tumour growth [132,133,134,135].

**Table 2:** Functional effects of complement on immune cells which promote tumour growth in mouse models.

Immune Cell	Model	Component	Observation	Mechanism	Ref
MDSC CD8^+^ T cell	Ovarian cancer, syngeneic (TC-1 cells)	C5a, C5aR	Tumour growth is impaired in C5aR^−/−^ mice Pharmacological blockade of C5aR reduces tumour growth	Recruitment of PMN MDSCs to tumours and production of ROS/RNS by MO MDSCs, suppresses CD8^+^ T cell responses	[16]
CD8^+^ T cell	Melanoma, syngeneic (B16 cells) Breast cancer, syngeneic (E0771)	C3, C3aR C5aR	Tumour growth is impaired in C3^−/−^ mice C3aR and C5aR antagonism reduces tumour growth.	Complement signalling inhibits IL-10 expression by CD8^+^ tumour-infiltrating lymphocytes (TILs), hindering the anti-tumour response	[136]
CD8^+^ T cell	Breast cancer, syngeneic (4T1 and 4TI-GFP cells)	C5a, C5aR	Reduced lung and liver metastases in C5aR^−/−^ mice C5aR antagonism reduces lung metastases	Recruitment of MDSCs, and induction of TGFB and IL-10 production, leads to suppression of CD8^+^ T cell function by Treg cells	[89]
CD8^+^ T cell	Lung cancer, syngeneic Kras^LSL-G12D/+^ mice (393P cells)	C5aR	Decreased tumour volume in C5aR^−/−^ mice Pharmacological blockade of C5a and PD-1 impairs tumour growth	Fewer MDSCs accompanied by an increase in CD8^+^ T cells, which had lower levels of exhaustion markers	[137]
CD8^+^ T cell	Colon cancer, syngeneic (MC38)	C3	Complement (C3) depletion using cobra venom factor (CVF) impairs tumour growth	C3 contributes to the generation of an immunosuppressive environment (Increased MDSCs, fewer CD8^+^ T cells, lower expression of CCL5, CXCL10 and CXCL11)	[138]
MDSC CD8^+^ T cell	Colitis-associated colorectal cancer (Induced by azoxymethane and dextran sulfate sodium)	C5aR	Tumour growth is impaired in C5aR^−/−^ mice C5aR antagonism reduces tumour growth	C5a recruits MDSCs to CRC tissue, inhibiting CD8^+^ T cell responses	[139]
CD4^+^ T cell CD8^+^ T cell	Lymphoma, syngeneic (RMA-3CF4 and RMA-1474 cells)	C5a	Tumour growth is impaired in mice with lymphoma cells producing low C5a levels	Increase in effector (IFN-y producing) CD4 and CD8^+^ T cells	[140]
CD4^+^ T cell	Lung cancer, syngeneic and orthotopic (LLC-luc, CMT-luc and EML4-ALK cells)	C3, C3aR, C5aR	Tumour growth is impaired and metastases are reduced in C3^−/−^ mice C3aR or C5aR antagonism reduces tumour growth.	Signalling of C3 prevents cytokine production by CD4^+^ T cells	[141]
MDSC	Lung cancer, syngeneic (3LL cells)	C5a, C5aR	C5aR antagonism reduces tumour growth	C5a contributes to the generation of an immunosuppressive microenvironment	[91]
MDSC	Hepatocellular carcinoma, syngenic (H22 cells)	C3	Tumour growth is impaired in mice with C3^−/−^ hepatic stellate cells	Hepatic stellate cells produce C3 leading to MDSC accumulation and immunosuppression	[142]
Neutrophil	Small intestine tumorigenesis (APC^Min/+^ mice)	C3aR	Tumour growth is impaired in C3aR^−/−^ mice	Engagement of C3aR on neutrophils drives NETosis and coagulation pathways to induce pro-tumorigenic low density neutrophils	[143]
Neutrophil	Colitis-associated colorectal cancer (Induced by azoxymethane and dextran sulfate sodium)	C3, C5, C5aR	Tumour growth is impaired in C3^−/−^, C5^−/−^ and C5aR^−/−^ mice	C5a induces neutrophil infiltration and IL-1B expression which drives IL-17A production	[144]
Neutrophil	Melanoma, syngeneic (B16F10)	C3aR	Tumour growth is impaired in C3aR^−/−^ mice C3aR antagonism arrests growth of established tumours	C3aR signalling reduces infiltrating neutrophils and CD4^+^ T cell populations	[145]
Macrophage	Melanoma, syngeneic (B16F10)	C3a, C3aR	C3a neutralization impairs tumour growth	C3a recruits macrophages which suppress the CD8^+^ T cell response	[146]
Macrophage	Sarcoma (Induced by 3-methylcholanthrane)	PTX3, C5a	PTX3 controls complement activation by recruiting Factor H. *Ptx3*^−/−^ mice are more susceptible to carcinogenesis	In the absence of PTX3, C5a generation is uninterrupted. An increase in CCL2 skews macrophages to an M2 phenotype	[147]
Macrophage	Colon cancer (metastatic), syngeneic (SL4 cells) Colon cancer xenograft (HCT116 and SW116 cells)	C5a, C5aR	Growth of hepatic metastases is impaired in C5aR^−/−^ mice or when C5 is downregulated or targeted via pharmacological blockade	C5a induces MCP-1 production by macrophages via the Akt pathway and promotes an immunosuppressive microenvironment	[148]
Macrophage	Pancreatic neuroendocrine tumours, transgenic (BT2B6)	C5aR	C5aR antagonism reduces tumour growth.	Increased infiltration of macrophages	[149]
Macrophage	Colon cancer, syngeneic (SL4-luc)	C5aR	Growth of hepatic metastases is impaired in C5aR^−/−^ mice	C5a polarises tumour associated macrophages (TAMs) to an M2 phenotype via NF-kB signalling	[150]
Macrophage and Mast cells	Squamous cell carcinoma, transgenic (K14-HPV16)	C5aR	Tumour growth is impaired in C5aR^−/−^ mice	C5aR signalling activates macrophages and mast cells, promoting a pro-tumour microenvironment and limiting CD8^+^ T cell responses	[151]
Natural Killer cell	Melanoma, syngeneic (B16gp33 cells)	C3	Complement (C3) depletion using CVF impairs tumour growth	Complement limits natural killer (NK) cell-mediation of the CD8^+^ T cell anti-tumour immune response	[152]
Natural Killer cell	Melanoma, syngeneic (B16-luc cells)	CR3	Metastases were reduced in CD11b^−/−^ (CR3 deficient) mice and mice with CR3 deficient NK cells	Interaction of iC3b with CR3 suppresses NK cells by activating SHIP and JNK pathways	[153]

#### 3.3.2. Human Studies

In humans, the expression of complement components is typically associated with adverse features and poor outcomes [91,100,107,154]. At the gene level, mutations, in particular driver mutations, as well as alterations and deletions in complement system genes are prevalent across at least 32 cancer types including lung, pancreatic and haematological malignancies [104]. The functional impact of these on cancer progression is further supported by the correlation between groups of complement mutations and survival; for example, the association of complement mutations with poor OS in low grade glioma [104]. Altered protein expression of complement components has also been described. In gastric cancer, tumours exhibit enhanced deposition of C3 and C3a relative to adjacent healthy tissue, with high C3 deposition observed to correlate with worse 5 year OS [155]. Evidence suggests that C3 deposits may activate JAK2/STAT3 to enhance tumour growth [155]. Others have observed increased C5aR expression and phosphorylated PI3K/AKT in gastric cancer tissue, when compared to matched normal tissue, with *in vitro* studies demonstrating C5a activation of PI3K/AKT [156]. Together, these studies provide evidence for complement-driven proliferation of gastric tumours. In breast cancer, expression of C5aR is associated with larger tumours, metastases in the lymph nodes and advanced clinical stages [14]. Furthermore, patients with C5aR negative tumours had improved survival rates when compared to those with C5aR positive tumours. Supporting this, C5a was demonstrated to promote proliferation of breast cancer cell lines, suggesting a role for complement signalling in breast cancer progression [14].

Conversely, complement has also been correlated with favourable clinical outcomes, suggesting a role for protection against tumour growth. High C3 levels are indicative of good prognosis in NSCLC, with greater numbers of infiltrating CD4^+^ and CD8^+^ T cells reported in tumours with increased C3 expression [157]. The differing prognostic implications of complement expression in tumour tissues demonstrate that complement functions in a context-dependent manner. This is likely due to the heterogenous TMEs that exist across cancer types and between cancer patients. The most recent transcriptomic analysis of complement genes expressed in cancer demonstrated that while there is little heterogeneity in whether complement genes are expressed by different cancer types, the heterogeneity of the specific complement genes expressed is great [19]. There is no doubt that the context-dependent nature of complement in facilitating tumour growth will have implications for identifying novel therapeutic approaches to target the complement cascade, but also in patient responses to first line therapies and immunotherapies.

## 4. Role of the Complement System in the Response to Cytotoxic Therapy

The many documented interactions between complement components and the TME highlight the potential of complement to induce alterations in immune cell function, local vasculature and the proliferative capability of tumour cells, all recognised hallmarks of cancer. These hallmarks also impact on the efficacy of traditional anti-cancer therapies such as chemotherapy and radiotherapy, as well as novel approaches such as immunotherapy. As such, it is unsurprising that emerging evidence demonstrates a role for complement in the response to anti-cancer therapy.

### 4.1. Complement and the Response to Radiotherapy

Radiation therapy (radiotherapy) is a major cancer treatment modality, received by over 50% of cancer patients [158]. The number of patients requiring radiotherapy is estimated to increase by 16% by the year 2025, based on projected cancer cases [159]. Understanding the molecular biology and TME related factors responsible for response to radiation is vital to optimise individual treatment regimens and to ensure therapeutic efficacy. Several studies in the literature demonstrate a relationship between radiation and the complement system, with recent evidence suggesting a role for complement in the tumour response to radiotherapy [17,160].

Radiotherapy is now understood to be an immunogenic process which initiates both innate and adaptive immune responses, however, control of tumour growth is primarily achieved via direct cell killing [161,162]. Irreparable DNA damage induced by ionising radiation causes tumour cell death via apoptosis and mitotic catastrophe, or cell cycle arrest leading to senescence [162]. Elvington et al. hypothesised that inhibiting complement would reduce complement-mediated clearance of apoptotic cells resulting in increased inflammation and necrotic cells, and a more immunogenic environment [17]. In a murine model of lymphoma, they demonstrated that complement inhibition in combination with radiotherapy significantly reduced the tumour growth rate, decreased tumour burden and improved survival when compared to radiotherapy alone [17]. Although complement activation is an inflammatory process, in this model, inhibition of complement in combination with radiotherapy promoted inflammation, when compared to radiotherapy alone. This was characterised by increased levels of IFN-γ, IL-6 and IL-17 [17]. Furthermore, early neutrophil infiltration followed by later infiltration of mature dendritic cells (DCs) and CD8^+^ T cells was observed, resulting in an enhanced anti-tumour immune response [17]. Ultimately, targeting complement improved therapeutic efficacy, suggesting that the interactions between complement and the TME can alter response to radiotherapy [17].

It is well established that radiotherapy induces immunogenic cell death and promotes anti-tumour immunity by enhancing T cell priming and effector phases [161,163,164,165]. The earliest step triggered by radiation that is responsible for initiating an immune response, however, is unclear. A potential mechanism involving complement was uncovered by Surace et al., who demonstrated that radiation activated the complement system, producing anaphylatoxins, which were essential for the subsequent response to radiotherapy [160]. In both mouse and human tumours, the classical and alternative complement pathways were activated following treatment with radiation. Radiotherapy failed to control tumours in mice deficient in either C3, C3aR or C5aR, indicating a functional role for complement in achieving treatment efficacy [160]. Analysis of infiltrating immune cells from irradiated and unirradiated tumours demonstrated that radiation induced complement expression in DCs and upregulated the expression of the C3aR and C5aR [160]. In line with previous reports, complement signals were essential for DC activation [85]. The functional importance of complement was also demonstrated in mice deficient in the C3aR and C5aR, with CD8^+^ T cells producing less IFN-γ post-radiation when compared to controls [160]. Independent of radiation, tumours from mice lacking the expression of complement receptors were characterised by increased numbers of regulatory T cells (Tregs) when compared to controls. These data demonstrate that in this model, complement is essential for activating DCs and promoting an efficient anti-tumour CD8^+^ T cell response [160]. When radiation was combined with the use of dexamethasone, a glucocorticoid that inhibits complement activation, the therapeutic effect of radiation was abolished, highlighting further the essential role for complement in the response to radiotherapy [160].

From these studies by Elvington et al., and Surace et al., it seems that two contradictory roles for complement may exist, with both inhibition and activation of complement demonstrated to enhance the tumour response, respectively [17,160]. There are several differences between the two studies which may explain these opposing results. Firstly, different murine models are utilised, a syngeneic model of lymphoma [17] and a syngeneic melanoma model [160]. Similar to the heterogeneity observed between human cancer types at a molecular and TME level, these tumour types will likely interact with immune cells and respond to treatment regimens differently. Notably, the response-enhancing effects of complement inhibition in a model of lymphoma was validated in a breast cancer model also [17]. Different methods of achieving complement inhibition were also used in each study. Elvington et al. used CR2-Crry, a complement inhibitor which directly targets sites where complement has been activated [166], while dexamethasone was used by Surace et al. [160]. Although dexamethasone inhibits complement activation [167] there is evidence that it can enhance the production of complement effectors under some circumstances such as high levels of IL-1α [168,169,170]. Importantly, the authors of these studies utilised different radiation regimens. Elvington et al. irradiated tumours using fractionated doses of radiation, which resulted in predominantly apoptotic cell death [17]. The bolus dose of radiation administered by Surace et al. induced necrosis and enhanced the accumulation of CD8^+^ T cells [160]. In these studies, fractionated radiotherapy in combination with complement inhibition [17] or single high dose radiotherapy without complement inhibition, efficiently controlled tumour growth [160], respectively. The dosage and method of administration of radiotherapy may be important. There is evidence that fractionated and ablative radiation doses have different effects on the immune system. Some studies demonstrate that single high doses of radiotherapy induce superior anti-tumour immunity, when compared to fractionated doses [171,172]. Interestingly, fractionated regimens are reported to generate efficient CD8^+^ T cell immune responses when combined with anti-CTLA-4 or anti-PD-1/PD-L1 immunotherapies [173,174,175]. It is possible that the chronic inflammation and increased complement activation induced by fractionated radiation limits the effector function of CD8^+^ T cells, as complement impairs effector CD8^+^ T cell function [16,89,136,137,138,140]. Further study of ablative versus fractionated radiotherapy regimens and their effects on immunogenicity and the activation of complement is required to understand how complement impacts the response to radiotherapy. However, together these two studies highlight that radiation induces local complement activation, which subsequently interacts with the TME to influence immune cell phenotype and alter the radioresponse.

### 4.2. Complement and the Response to Chemotherapy

As for radiation therapy, chemotherapeutic agents may alter the TME and enhance the immunogenicity of tumours by inducing immunogenic cell death. The interactions between complement and local immune cells may have a key role in this process. Medler et al. identified a role for macrophage-produced C5a in squamous cell carcinogenesis, whereby signalling through the C5aR activates mast cells and macrophages, promoting a pro-tumour, immunosuppressive microenvironment [151]. They demonstrated, in a murine model, improved tumour response to paclitaxel (PTX) chemotherapy following treatment with a C5aR antagonist, PMX-53. The combination of PTX and PMX-53 resulted in transcriptional reprogramming of tumour-associated macrophages and subsequent recruitment of CXCR3^+^ effector and memory CD8^+^ T cells to significantly reduce tumour burden, when compared to treatment with PTX alone [151]. This suggests that C5a signalling remodels the TME by restricting CD8^+^ T cell infiltration. Inhibiting complement within the TME of SCCs therefore may have potential for improving response to chemotherapy.

More recently, a role for complement signalling in the response to chemotherapy in breast adenocarcinoma was identified. Transcriptomic analysis of intratumoural B cells highlighted an inducible T cell costimulatory ligand (ICOSL) positive B cell population, which was enriched following initiation of neo-adjuvant chemotherapy, when compared to pre-treatment [176]. In agreement, murine studies demonstrated a doxorubicin-induced increase in ICOSL^+^ CR2^+^ B cells [176]. Importantly, ICOSL^+^ B cells were of clinical significance, correlating with improved DFS and OS, and were also associated with complement activation. Chemotherapy-associated immunogenic cell death was demonstrated to induce complement activation. Complement activation fragments bound CR2, promoting B cell switching to an ICOSL^+^ phenotype. In patients with tumours overexpressing CD55, the accompanying reduction in complement activation correlated with chemoresistance and poorer outcomes [176]. Complement largely appears to protect against tumour growth in breast adenocarcinoma [132,133,134,135]. These data support that in this cancer type, complement activation has an anti-tumour function and is essential for chemotherapeutic efficacy [176]. Further study of the impact of ICOSL^+^ B cells in the TME is required to determine their relevance in the response to therapy of other cancers [177].

Complement has also been demonstrated to alter response to chemotherapy independent of immune cells. Endometrioid tumours overexpress CD55 relative to benign tissue [178]. This expression is associated with resistance to cisplatin chemotherapy, with CD55 positive cells exhibiting markedly increased self-renewing ability when compared to CD55 negative cells [179]. In particular, CD55 is highly expressed by cancer-stem cells and cisplatin-resistant cancer cells. Functional studies have demonstrated that CD55 localises to lipid rafts to activate ROR2-JNK and LCK pathways [179]. Signalling through these pathways drives self-renewal and resistance to cisplatin, respectively. This tumour-promoting role for CD55 is in contrast with that elucidated by Lu et al., whereby expression of CD55 inhibits complement activation required for the B cell response to chemotherapy in breast cancer [176]. Together, these studies provide evidence of context-dependent roles for complement in the response to chemotherapy.

### 4.3. Potential Mechanisms Underlying Complement-Mediated Resistance to Cytotoxic Therapy

#### 4.3.1. Hypoxia

In addition to an altered immune milieu within the TME, one of the greatest barriers to achieving therapeutic efficacy in the treatment of solid tumours is hypoxia. Hypoxia refers to regions that are oxygen deprived (<2% O_2_), which in tumours arise due to the rapid proliferation of neoplastic cells and disorganised vasculature [180]. Within hypoxic areas, hypoxia-inducible factors (HIFs) drive the expression of pro-survival genes, promoting tumour progression through activation of angiogenesis and metastasis [181,182]. Tumour hypoxia is a major challenge to radiotherapy, given that oxygen is essential for the generation of DNA-damaging free radicals during treatment [183,184,185]. Hypoxic cancer cells are three times less sensitive to killing by radiation, when compared to oxygenated cancer cells, and as a result hypoxia is associated with poor patient prognosis and treatment outcomes [186,187,188]. Additionally, chemosensitivity is impaired under hypoxic conditions [189,190]. In this context, hypoxia has been demonstrated to induce activation of all three complement pathways [191]. Olcina and colleagues recently demonstrated in colorectal cancer that mutations in complement genes were associated with enrichment of hypoxia gene signatures and poor OS [104]. This suggests that complement may contribute to the development of a hypoxic TME, potentially having a negative impact on patient outcomes [104]. They also demonstrated HIF-dependent upregulation of CD55, which promoted resistance to CDC [104]. Thus, there appears to be bidirectional crosstalk between complement and hypoxia in colorectal cancer [104].

Direct evidence for altered treatment response as a result of hypoxia-mediated regulation of complement has not been reported. At present, the literature demonstrates the complexity of this relationship and its potential to modify the TME, with evidence that exposure to hypoxia induces alterations in complement gene expression [192,193,194]. Further investigation of the hypoxia-complement interplay will yield insights into how this may be impacting therapeutic efficacy.

#### 4.3.2. DNA Repair

In addition to hypoxia, alterations in DNA repair capabilities have been associated with poor response to standard cancer therapies. The DNA damage response (DDR) is essential to preserving genomic integrity. Recognition of compromised cellular DNA initiates the DDR, leading to damage repair or activation of downstream pathways to halt cell cycle progression or induce apoptosis [195]. The progression from a pre-cancerous to neoplastic cell is enabled by increased genomic instability, with cancer cells acquiring defects in, or loss of, DDR pathways [10,196]. Dependence on fewer repair pathways is exploited by conventional radiotherapy and some chemotherapies, which induce lethal DNA damage, and also by newer targeted therapies which can inhibit specific DDR pathways, making it difficult for cells to repair damage [197]. Despite this, we and others have demonstrated that cancer cells displaying resistance to treatment possess more efficient DNA repair strategies [198,199,200]. Previously, our group demonstrated that C3 mRNA expression was significantly increased in tumour biopsies of oesophageal adenocarcinoma patients with a subsequent poor response to neoadjuvant chemoradiotherapy (neo-CRT) [201]. Global micro-RNA (miR) profiling revealed that miR-187 was significantly decreased in these tissue samples. Similarly, in a miR-187 overexpressing oesophageal adenocarcinoma (OAC) cell line, C3 mRNA was downregulated, suggesting negative regulation of C3 by miR-187. Interestingly, these *in vitro* studies demonstrated that overexpression of miR-187 sensitised OAC cells to both radiotherapy and cisplatin and this was accompanied by downregulation of several DDR genes. While the mechanism of action by which miR-187 expression alters response to treatment remains to be elucidated, its regulation of C3 expression and DDR genes suggests that complement may interact with DDR genes to influence response to therapy.

Genetic polymorphisms in DNA repair genes impact on carcinogenesis and therapeutic resistance. The DNA repair protein x-ray repair cross complementing 3 (XRCC3) is a key participant in homologous recombination of DNA double-strand breaks [202]. Genotype variants at the rs1861539 polymorphic site of the XRCC3 gene result in defective DNA repair and are associated with increased risk for several cancers including lung and childhood acute lymphoblastic leukemia [203]. Low serum levels of complement C3 and C4 have been correlated with specific genotypes of the *XRCC3* gene at this site, when compared to the wildtype genotype [204]. While this research investigated styrene-exposed individuals, it would be interesting to repeat in the setting of cancer. Elucidating the potential relationship between altered complement expression levels and polymorphisms in DNA repair genes may provide new therapeutic perspectives.

#### 4.3.3. Metabolism

Successful propagation of malignant tumours is generally accompanied by altered energy metabolism, a hallmark required to fuel the rapid growth demands of cancer [10]. The Warburg effect refers to the preferential use of glycolysis over oxidative phosphorylation (OXPHOS) by cancer cells, even in the presence of oxygen [205,206]. Modifications in cellular metabolism are associated with resistance to most cancer therapeutics, including targeted inhibitors and adopted cell therapy [207,208,209,210,211]. Metabolism, and the drivers of these metabolic alterations therefore represent potential therapeutic targets to improve treatment response. Considering the roles played by the complement system in homeostasis, it is unsurprising that complement components also function in metabolism, in particular, in T cells, a subject recently reviewed [212,213,214]. Autocrine complement signalling is essential for the survival of naïve T cells and optimal T cell expansion following activation [85,86,116]. Intracellularly, complement signals orchestrate Th1 effector responses [115,116,215], the induction of which are characterised by complement-driven metabolic reprogramming of CD4^+^ T cells [117]. Interaction of C3b with CD46 drives metabolic events to induce the Th1 response. Upregulation of glucose and amino acid transporters facilitates nutrient uptake, while LAMTOR5 expression increases glycolysis and OXPHOS [117]. Complement signalling therefore joins antigen recognition, co-stimulation and cytokines as an important modulator of T cell differentiation. Unfortunately, patients with a poor response to cancer therapy have poorly infiltrated tumours, with evidence of phenotypically exhausted or dysfunctional effector T cells [216]. Given the increasing evidence for dysregulated complement expression within the TME, it is likely that perturbations in the levels of complement components impact T cell metabolism and effector induction. Further study is necessary to understand the potential impact on patient response to treatment.

#### 4.3.4. PI3K/Akt Signalling

The phosphatidylinositol 3 kinase (PI3k)/Akt signalling pathway is a key regulator of several cell death and survival mechanisms [217]. Frequently mutated in cancer, this pathway is exploited by cancer cells to undergo accelerated growth and resist apoptosis, often rendering them treatment-resistant [218]. In renal cell carcinoma, the C5a/C5aR axis has been demonstrated to activate PI3K and extracellular signal-related kinase (ERK) signalling pathways, promoting invasiveness and metastasis [125]. Similarly, in ovarian cancer cells, signalling through anaphylatoxin receptors plays a pro-tumorigenic role by enhancing proliferation [118]. Evidence for complement-mediated activation of PI3K/Akt suggests that these signals may allow cancer cells to resist treatment through downregulation of apoptotic pathways, a known mechanism of treatment resistance. The MAC has also been demonstrated to control cell survival. Sublytic insertion of MACs induces PI3K/Akt pathway activation and inhibits apoptosis in oligodendrocytes [219]. Many studies have reported deposition of MAC in various cancers [99]. It is possible that these deposition levels may trigger PI3K/Akt signalling and contribute to poor therapeutic responses. This axis may be another opportunity for complement to modulate cancer cells and reduce treatment efficacy. Further characterisation of complement-mediated activation of PI3K/Akt signalling pathways is required to determine if this contributes to patient response to treatment in cancer.

#### 4.3.5. Exosomes

The transport of biological material by extracellular vesicles (EVs) has emerged as an important mechanism of intercellular communication [220]. Exosomes are small EVs of endosomal origin, which range in size from 30–150 nm [221,222]. All cells secrete exosomes including immune [223] and cancer cells [224], and they are present in bodily fluids such as blood, saliva and cerebrospinal fluid [225,226]. Structurally, exosomes consist of a phospholipid bilayer, which encloses protein and nucleic acids derived from the cell of origin [227]. Exosome contents can have effects on target cells through fusion with the plasma membrane and subsequent delivery of their cargo to the recipient cell or by binding to cell surface receptors [228]. This intercellular transfer of information by exosomes has many functionally important roles in normal tissue physiology [229]. Increasing evidence demonstrates that exosomes and their contents also play roles in cancer [228,230,231]. Exosomes appear to largely play a tumour promoting role [224,232] and have been associated with immunosuppression within the TME [233,234] and the development of pre-metastatic niches [235,236]. Complement components are among the many proteins associated with exosomes, a subject recently reviewed in detail [237]. Briefly, complement proteins have been identified in exosomes secreted from numerous cell types including antigen-presenting cells [238,239] and cancer cells [240,241]. Tumour cells can also eliminate surface MAC complexes by shedding microvesicles, allowing them to escape from complement-mediated lysis [242,243].

Increasing evidence suggests that exosomes play a role in treatment resistance, both by acting as a decoy for immunotherapies and by exporting drugs from cancer cells [244,245]. A recent proteomic analysis has demonstrated that complement components including C3 and CD55 are among the proteins enriched in exosomes derived from cisplatin resistant lung cancer cells, relative to cisplatin sensitive cells [241]. As previously discussed, emerging evidence has indicated that CD55 is associated with resistance to chemotherapy [176,179]. Together, these data suggest that exosome-associated mCRPs potentially engage in roles that influence response to cancer treatment. There is great interest in understanding the immunosuppressive mechanisms within the TME responsible for therapeutic resistance. Exosomes derived from ovarian tumours have been demonstrated to induce T cell arrest [246]. This has been linked to the presence of phosphatidylserine, an activator of the classical complement pathway, on the surface of these tumour-derived exosomes [246]. Further investigation of complement signals driven by exosome-associated complement components or ligands is necessary to determine whether this may promote immunosuppression within the TME. In patients with sepsis, neutrophil dysfunction is associated with shedding of the C5aR, potentially via EVs [247]. In the context of cancer, investigation of the shedding of complement receptors by immune cells is necessary to determine whether exosome-driven loss of receptors may also be influencing immune dysfunction. Given the roles for complement in immune cell activation [248], the presence of mCRPs on tumour-derived exosomes may additionally have effects in coordinating the immune response [237].

Although studies demonstrating a role for complement in exosome-associated therapeutic resistance are limited, the body of research is growing [237]. For example, we now understand that in B-cell lymphoma, complement may be consumed by exosomes to protect against anti-CD20 antibodies [244]. It is likely that the impact of exosome-mediated signalling in cancer is far greater than currently understood. Further investigation of the presence of complement components in exosomes in a range of cancer types, and the functional impact of these within the TME is required to understand if exosome-associated complement alters response to treatment.

### 4.4. Complement as A Biomarker of Response to Cytotoxic Therapy

There is global interest in identifying biomarkers of response to chemotherapy, radiotherapy or combined chemoradiation therapy (CRT) with the aim of reducing treatment-associated toxicities and ensuring optimal treatment strategies for patients.

The expression of several complement cascade genes has recently been correlated with the chemosensitivity of soft tissue sarcomas (STS) [249]. Analysis of TCGA by Zhang et al. indicated that several genes were differentially expressed between STS subtypes with varying chemosensitivities, many of which coded for complement system components. Those STS that were relatively insensitive to chemotherapy treatment expressed high levels of complement system genes, and this had clinical significance [249]. High expression of the C3aR, C1QC and FI correlated with poor OS. Upregulation of C3aR was also associated with worse DFS, suggesting that these complement components play a role in the tumour response to chemotherapy in STS [249]. These genes thus represent novel biomarkers of tumour chemosensitivity and patient survival in STS.

In breast cancer, resistance to neo-adjuvant chemotherapy remains a clinical challenge. Proteomic analysis of human plasma from breast cancer patients has demonstrated that complement is modulated by epirubicin and docetaxel, with alterations in complement components reported as early as 24 h following the initiation of treatment [250]. Interestingly, the levels of C3 isoforms differed between responders and non-responders. As the immediate response in plasma correlated with the final tumour response to treatment, this suggests that C3 isoforms may have potential as early predictive biomarkers of response to epirubicin and docetaxel in breast cancer [250].

We have performed proteomic profiling of pre-treatment serum samples from oesophageal cancer patients and reported that increased C3a and C4a levels predict a subsequent poor response to neo-CRT [251]. Our study was the first to implicate these anaphylatoxins in the response to neo-CRT. In support of this, using pre-treatment tissue, we demonstrated that oesophageal cancer patients with a subsequent poor pathological response to neo-CRT had increased tumoural expression of C3, when compared to good responders [201]. This work suggests that complement may have potential as a tumoural and/or circulating biomarker of response to treatment. To determine the full potential of complement components as predictive markers of therapeutic response, further validation studies encompassing more cancer types and treatment regimens are required.

## 5. The Implications of the Complement System and Immunotherapy

The cancer immunotherapy field has celebrated many notable milestones in the last two decades, with clinically approved immunotherapies greatly improving therapeutic options for patients with metastatic cancer, most notably melanoma and NSCLC [252]. These treatments take advantage of the host immune system by overcoming suppression of immune cells or boosting their effector potential. In addition, cancer vaccines, adoptive cell therapy, oncolytic viruses and monoclonal antibodies are all among the treatment regimens used to exploit natural defence mechanisms and augment anti-tumour immunity [253,254].

Monoclonal antibody (mAb) therapy is arguably the most successful branch of immunotherapy at present. Several mAbs have been approved for the treatment of solid and haematological malignancies following clinical demonstration of impaired disease progression and improved patient survival [255,256]. Various tumour antigens and tumour promoting signalling pathways can now be targeted by mAbs, with activity of the complement system essential for mAb-induced cytotoxicity. Complement plays a role in the cytotoxicity of mAbs which target B cell antigens, including rituximab and daratumumab and also trastuzumab, a HER2 specific mAb [257,258,259,260]. Binding of mAbs to specific tumour cell antigens may initiate CDC, antibody-dependent cellular cytotoxicity (ADCC) or aid phagocytosis in a mechanism referred to as complement-dependent cell-mediated cytotoxicity (CDCC) [261,262,263]. Fc regions of Igs bound to tumour cells interact with C1q, inducing activation of the complement cascade and MAC-mediated cell lysis (CDC). This functional contribution of complement towards the cytotoxicity of mAbs has influenced their design. Many clinically approved mAbs are of the IgG class, in particular IgG1 and IgG4, as C1q is effective at fixing these isotypes [264]. Activation of the complement system further contributes to the efficacy of mAbs by aiding phagocytic cells. Phagocytosis of malignant cells is enhanced due to opsonisation of cells by complement fragments such as C3b which interact with CR3/CR4 expressed by phagocytes [265,266]. The Fc portion of mAbs can also interact with activating FcγRs of natural killer (NK) cells, macrophages and neutrophils recruited by complement anaphylatoxins, to induce ADCC [267,268,269,270].

### 5.1. Immune Checkpoint Inhibition

Among the most promising of mAb therapies are immune checkpoint inhibitors [271]. These drugs target immune cell receptors or ligands that engage in inhibitory interactions and downregulate immune responses. Most notable are cytotoxic T lymphocyte-associated protein 4 (CTLA-4) and programmed death-1 (PD-1) expressed by activated T and B cells [272,273]. When PD-1 expressed by T cells binds its ligands PD-L1 or PD-L2, T cell receptor signalling is impaired, consequently limiting proliferation and inhibiting cytokine secretion [274]. This interaction therefore plays a key role in maintaining T cell homeostasis [275,276]. Expression of PD-L1 by cancer cells provides a mechanism for tumours to downregulate the immune response, escaping elimination by cytotoxic effector cells [277]. Currently three anti-PD-1 mAbs are approved for the treatment of specific cancer types [278,279,280]. PD-1 blockade is successful in restoring effector potential of dysfunctional T cells [281]. Although effective in susceptible patient cohorts, primary and acquired resistance is a clinical challenge to anti-PD-1 and anti-PD-L1 therapy [282,283].

#### Combination Inhibition of Complement and PD-1 Signalling

Combinations of immune therapies are proposed to counter resistance [284]. Complement may have a role in treatment response as it suppresses local immune cells (Table 2) and is associated with resistance to anti-PD-1 therapy in melanoma [285]. In several studies, a relationship between complement components and expression of immune checkpoints has been demonstrated [15,91,286]. In clear-cell renal cell carcinoma, C1q expression has been correlated with expression of PD-L1, PD-L2, PD-1, lymphyocyte-activation gene 3 (LAG3), T cell immunoglobulin and mucin-domain containing 3 (TIM3) and CTLA4 expression, suggesting that complement may contribute to T cell exhaustion [15]. In a murine model of lung cancer, C5a antagonism results in decreased expression of PD-L1, LAG3 and CTLA4, again highlighting that complement signalling potentially controls the T cell phenotype within the TME [91]. C5a has also been reported to induce PD-L1 expression in a whole blood model [286]. Considering these data, the complement system therefore appears an attractive target to overcome immunosuppression. In order to improve patient responses, complement inhibition has been proposed as a potential addition to immunotherapy [138,287,288].

Several studies have interrogated the effects of targeting both complement and PD-1/PD-L1 signalling. Wang et al. demonstrated using murine melanoma and breast cancer models that complement signalling in CD8^+^ T cells inhibits IL-10 production, reducing the anti-tumour immune response [136]. Although IL-10 has widely been regarded as an immunosuppressive mediator, recent clinical studies have indicated that IL-10 can re-invigorate CD8^+^ T cell responses [289,290]. Interestingly, C3aR and C5aR antagonism in combination with PD-1 blockade demonstrated superior inhibition of melanoma tumour growth, when compared to either treatment alone [136]. The anti-tumour effect of targeting both complement and PD-1 suggests that dual blockade of these pathways may synergise to overcome immunosuppression [136].

This synergism has also been demonstrated in murine models of lung cancer, with combined C5aR and PD-1 inhibition demonstrated to induce greater tumour regression relative to monotherapy [137]. Depletion of MDSCs in addition to PD-1 blockade achieved control of tumour growth similar to combination complement and PD-1 inhibition [137]. Importantly, targeting both complement and PD-1 lowered expression of exhaustion markers by CD8^+^ T cells [137]. These data suggest that complement signalling is a driver of immunosuppression within the TME, inhibiting the C8^+^ T cell response and potentially limiting therapeutic efficacy. Similarly, Zha and colleagues demonstrated that inhibition of C5aR signalling improves response to PD-L1 blockade in murine colon cancer and melanoma models [291]. They also highlighted that CD8^+^ T cells are essential to therapeutic efficacy and demonstrated that complement inhibition reduces MDSC-mediated immunosuppression [291].

**Figure 2:**
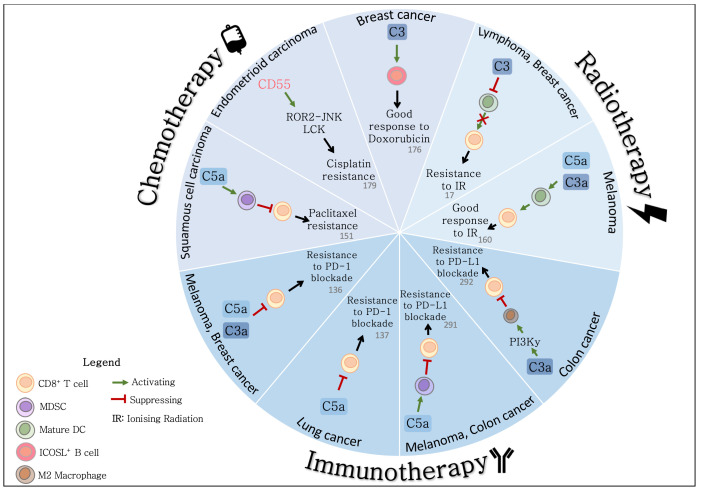
The influence of complement on response to treatment within the tumour microenvironment. The current understanding of the roles for complement in the response to radiotherapy, chemotherapy and monoclonal antibody-based immunotherapies are depicted. Green arrows illustrate events where complement component(s) have been demonstrated to activate an immune cell or signalling pathway, while inhibitory effects are shown in red. Black arrows indicate the observed influence of complement activities on the therapeutic efficacies of the designated chemotherapy, radiotherapy or immunotherapy treatment, in specified cancer types.

Complement regulation of macrophages has been demonstrated to reduce the efficacy of anti-PD-L1 mAbs. Signalling of tumour cell-derived C3a via C3aR expressed by tumour-associated macrophages (TAMs) promotes an M2 phenotype and suppresses effector CD8^+^ T cells in colon cancer [292]. Improved effectiveness of PD-L1 therapy has been demonstrated in C3-deficient tumours when compared to controls [292]. Collectively, these studies establish complement as a modulator of immune cells within the TME, which subsequently may affect response to radiotherapy, chemotherapy and immunotherapy treatment (Figure 2). A phase I trial (STELLAR-001) initiated by Innate Pharma is currently investigating an anti-C5aR drug in combination with durvalumab (anti-PD-1), with preliminary results suggesting that treatment toxicity is manageable [293].

On account of the complement-mediated regulation of IL-10 production by CD8^+^ T cells, it has been proposed that the C3a and C5a receptors represent novel immune checkpoints [294]. There are caveats, however. First, a greater understanding of autocrine complement signalling in CD8^+^ T cells is required to determine the full potential of complement in this regard. Essential roles for complement in the T cell life cycle and Th1 differentiation are well established [85,86,87,88]. These homeostatic functions contrast greatly with the observed negative influence of complement on T cell effector functions, as discussed in this review. Notably, these initial studies were focused on CD4^+^ T cells while investigations in the cancer setting studied CD8^+^ T cells [295]. Second, complement signals have been demonstrated to modulate regulatory T cells (Tregs). Signalling via C3aR and C5aR lowers forkhead box P3 (FoxP3) expression to limit Treg function [296]. As a result, in the absence of complement signals, Treg suppressive abilities are enhanced. There is potential that complement blockade may favour these suppressive T cells. It appears that complement effects differ depending on the disease setting and T cell subset, as once again, the context-dependent nature of complement signals become apparent. Due to the widespread expression of complement receptors, C3aR and/or C5aR blockade is likely to have diverse effects on the cellular component of the TME. Moreover, despite current evidence highlighting the potential therapeutic benefits of targeting complement signalling, complement is recognised to contribute to the efficacy of most mAbs. Manipulating complement signalling has the potential to restore an effective anti-tumour immune response, however the functional impact of this on mAb-induced cytoxicity remains to be determined. Finally, elucidating the full potential of combined PD-1 and complement receptor blockade requires investigation in a range of cancer stages and models.

### 5.2. Implications of CRPs for Immunotherapy

There is reasonable evidence to assume that incorporation of complement-targeted therapeutics into immunotherapy regimens may improve treatment response. One strategy is the neutralisation of mCRPs, overexpression of which have been observed to interfere with response to mAbs such as trastuzumab and rituximab [297,298,299,300]. Key stages of the complement cascade are inhibited by mCRPs, a technique cancer cells exploit to escape complement-mediated killing [301]. These regulators are a major obstacle to mAb-based therapy as they limit the degree to which CDC can contribute to mAb efficacy [103]. Blockade of mCRPs has been demonstrated to overcome resistance to mAb therapy by enhancing cytotoxic efficacy [297,298,299]. The relationship between mCRP expression and response to immune checkpoint inhibitors has not yet been investigated. It has been proposed that the use of neutralising antibodies against mCRPs may not be useful in the setting of PD-1/PD-L1 blockade as they would increase complement activation products such as C5a, which have been demonstrated to have a negative influence on this signalling axis [108]. Of mCRPs to target, CD59 blockade may have the potential to improve therapeutic efficacy and outcomes by increasing CDC [108]. The use of complement therapeutics to modulate mCRP function in combination with immune checkpoint inhibitors requires further understanding of the expression of mCRPs in cancers treated with these antibodies, and also, the degree to which they contribute to therapeutic resistance in this setting. Importantly, combining complement inhibition with immunotherapy warrants investigation of optimal scheduling in order to ensure that therapeutic efficacy is maximised.

### 5.3. Cancer Vaccines

Cancer vaccines are another branch of immunotherapy demonstrating considerable potential to revolutionise cancer therapeutics. In contrast with vaccines for microbial-associated diseases, a unique set of challenges are associated with cancer vaccine development including a typically immunosuppressive TME and the challenge of identifying a suitable tumour antigen to target [302]. One mechanism to circumvent the former of these issues is the use of vaccine adjuvants to stimulate and promote anti-tumour immunity. Complement system components play essential roles in coordinating the adaptive immune response, therefore it is unsurprising that they have been investigated as vaccine adjuvants to boost the immune response. Conformationally-biased agonists of C5a have previously been reported to enhance antigen processing and presentation by DCs and induce Th1-type cytokine production [303,304]. Conjugation of C5a with a live fungal vaccine has also demonstrated the potential of complement components as immune adjuvants, by polarising the T cell repertoire to a Th1/Th17 response, reducing histopathological inflammation and improving survival [305]. In the context of cancer, a C5a agonist linked to known tumour-antigens has been investigated in a murine model of melanoma, demonstrating a reduction in tumour growth and improved survival [306]. The potential of C5a-based agonists has also been demonstrated in a model of B cell lymphoma [307]. The proposed mechanism of action responsible for therapeutic efficacy is via the C5aR expressed by DCs and subsequent processing and presentation of tumour antigens to stimulate an anti-tumour immune response [306]. Further studies are required to fully elucidate the events occurring upon binding of C5a agonists to the C5aR and to characterise the antigen processing [306].

Evidence has also been provided for the potential of C3d-based adjuvants in cancer vaccines. While the roles played by antigen-bound C3d in B cell responses are well established, less is known about the function of free C3d in immunity. In murine lymphoma and melanoma models, vaccination with C3d^+^ killed cancer cells was associated with enhanced adaptive immunity and greater tumour control, when compared to unvaccinated and C3d^−^ vaccine controls [308]. This was characterised by enhanced T cell infiltration, apoptosis of Tregs and a reduction in PD-1 expression [308]. C3d has also been investigated as a molecular adjuvant to an anti-angiogenic (vascular endothelial growth factor receptor 2) vaccine, demonstrating impaired tumour growth and conferring a significant survival advantage [309]. Together, these studies demonstrate that complement components can enhance vaccine-induced anti-tumour immunity. Further research is required to determine the full potential of complement components as vaccine adjuvants including the specific interactions with local immune cells that lead to control of tumour growth.

## 6. Conclusions

Cancer is a disease that arises when cellular division deviates from homeostasis. Considering the recent discoveries that identify the complement system as a key component of many homeostatic processes, it could only be expected that a relationship between complement and cancer is beginning to emerge. Although roles for the complement system in cancer are often paradoxical, perhaps similar to the contrasting impact of acute compared with chronic inflammation on tumour pathogenesis, it appears that the complement system has context-specific properties with respect to anti-tumour immunity and response to cancer therapies. New knowledge from TCGA and other sources highlights that, although transcriptomic analysis may be useful in determining the potential prognostic significance that accompanies expression of complement genes, such analyses exclude key microenvironmental influences such as local production of anaphylatoxins by immune cells that have great relevance *in vivo* [19]. The integration of the complement system with many other cellular processes presents a challenge, with a limiting factor being the difficulty in deriving mechanistic evidence from patient biopsies and blood samples when compared to rodent models. Although rodent models have provided much information on this subject, heterogenous observations between cancer types and regarding the complement activation pathway responsible demonstrates that we may be just at the tip of the iceberg in elucidating the relationship between complement and cancer.

Despite these complexities in the elucidation of the role of complement in carcinogenesis, increasing evidence suggests that manipulation of complement holds significant promise in understanding resistance to standard and emerging cancer therapies, and in the development of novel therapies. Determining the true potential of complement as a companion therapeutic target certainly requires further understanding of the interactions between complement and the cellular and non-cellular components of the TME. In time, this will allow us to elucidate how harnessing these interactions can improve response to radiotherapy, chemotherapy and immunotherapy treatments.

## Figures and Tables

**Table 1:** Membrane-bound complement regulatory proteins.

Regulator	Alternative Name (s)	Distribution	Function	Reference
CD35	Complement receptor 1 (CR1)	Primarily lymphocytes, erythrocytes, phagocytes, dendritic cells	Cofactor for C3b and C4b degradation by Factor H Accelerates C3 and C5 convertases	[51,52,53,54,55]
CD46	Membrane cofactor protein (MCP)	All nucleated cells	Cofactor for C3b and C4b degradation by Factor H	[56,57,58]
CD55	Decay accelerating factor (DAF)	Ubiquitously expressed	Accelerates decay of C3 and C5 convertases	[59,60,61]
CD59	Membrane-inhibitor of reactive lysis (MIRL), MAC inhibitory protein (MAC-IP), Protectin	Ubiquitously expressed	Binds C5b-C9 to prevent polymerization of C9	[62,63]

## Abbreviations

C3aR, C3a receptor; C5aR, C5a receptor; CDC, complement-dependent cytotoxicity; CDCC, complement-dependent cell-mediated cytotoxicity; CR1, complement receptor 1; CR2, complement receptor 2; CR3, complement receptor 3; CR4, complement receptor 4; DC, dendritic cell; DFS, disease-free survival; FH, factor H; FI, factor I; HIF, hypoxia-inducible factor; MAC, membrane attack complex; MASP, mannose-binding lectin-associated serine protease; MBL, mannose-binding lectin; mCRP, membrane-bound complement regulatory protein; MDSC, myeloid-derived suppressor cell; MO, mononuclear; neo-CRT, neo-adjuvant chemoradiation therapy; OS, overall survival; PMN, polymorphonuclear; TIL, tumour-infiltrating lymphocyte; TME, tumour microenvironment; Treg, regulatory T cell.

## References

[1] Coussens L.M., Werb Z. (2012). Inflammation and cancer. Nature.

[2] De Visser K.E., Eichten A., Coussens L.M. (2006). Paradoxical roles of the immune system during cancer development. Nat. Rev. Cancer.

[3] Reis E.S., Mastellos D.C., Ricklin D., Mantovani A., Lambris J.D. (2018). Complement in cancer: Untangling an intricate relationship. Nat. Rev. Immunol..

[4] Ricklin D., Hajishengallis G., Yang K., Lambris J.D. (2010). Complement: A key system for immune surveillance and homeostasis. Nat. Immunol..

[5] Carroll M.C. (2004). The complement system in regulation of adaptive immunity. Nat. Immunol..

[6] Schifferli J.A., Ng Y.C., Peters D.K. (1986). The role of complement and its receptor in the elimination of immune complexes. N. Engl. J. Med..

[7] Nozaki M., Raisler B.J., Sakurai E., Sarma J.V., Barnum S.R., Lambris J.D., Chen Y., Zhang K., Ambati B.K., Baffi J.Z. (2006). Drusen complement components C3a and C5a promote choroidal neovascularization. Proc. Natl. Acad. Sci. USA.

[8] Stevens B., Allen N.J., Vazquez L.E., Howell G.R., Christopherson K.S., Nouri N., Micheva K.D., Mehalow A.K., Huberman A.D., Stafford B. (2007). The Classical Complement Cascade Mediates CNS Synapse Elimination. Cell.

[9] Pio R., Corrales L., Lambris J.D. (2014). The Role of Complement in Tumour Growth. Adv. Exp. Med. Biol..

[10] Hanahan D., Weinberg R.A. (2011). Hallmarks of Cancer: The Next Generation. Cell.

[11] Ytting H., Christensen I.J., Steffensen R., Alsner J., Thiel S., Jensenius J.C., Hansen U., Nielsen H.J. (2011). Mannan-Binding Lectin (MBL) and MBL-Associated Serine Protease 2 (MASP-2) Genotypes in Colorectal Cancer. Scand. J. Immunol..

[12] Storm L., Christensen I.J., Jensenius J.C., Nielsen H.J., Thiel S. (2015). Evaluation of complement proteins as screening markers for colorectal cancer. Cancer Immunol. Immunother..

[13] Kesselring R., Thiel A., Pries R., Fichtner-Feigl S., Brunner S., Seidel P., Bruchhage K.L., Wollenberg B. (2014). The complement receptors CD46, CD55 and CD59 are regulated by the tumour microenvironment of head and neck cancer to facilitate escape of complement attack. Eur. J. Cancer.

[14] Imamura T., Yamamoto-Ibusuki M., Sueta A., Kubo T., Irie A., Kikuchi K., Kariu T., Iwase H. (2016). Influence of the C5a–C5a receptor system on breast cancer progression and patient prognosis. Breast Cancer.

[15] Roumenina L.T., Daugan M.V., Noé R., Petitprez F., Vano Y.A., Sanchez-Salas R., Becht E., Meilleroux J., Clec’h B.L., Giraldo N.A. (2019). Tumor Cells Hijack Macrophage-Produced Complement C1q to Promote Tumor Growth. Cancer Immunol. Res..

[16] Markiewski M.M., Deangelis R., Benencia F., Ricklin- S.K., Koutoulaki A., Gerard C., Coukos G., Lambris J.D. (2008). Modulation of the anti-tumor immune response by complement. Nat. Immunol..

[17] Elvington M., Scheiber M., Yang X., Lyons K., Jacqmin D., Wadsworth C., Marshall D., Vanek K., Tomlinson S. (2014). Complement dependent modulation of anti-tumor immunity following radiation therapy. Cell Rep..

[18] Kourtzelis I., Rafail S. (2016). The dual role of complement in cancer and its implication in anti-tumor therapy. Ann. Transl. Med..

[19] Roumenina L.T., Daugan M.V., Petitprez F., Sautès-Fridman C., Fridman W.H. (2019). Context-dependent roles of complement in cancer. Nat. Rev. Cancer.

[20] Bordet J., Gengou O. (1901). Sur l’existence de substances sensibilisatrices dans la plu- part des serum antimicrobiens. Ann. Inst. Pasteur Paris.

[21] Nesargikar P.N., Spiller B., Chavez R. (2012). The complement system: History, pathways, cascade and inhibitors. Eur. J. Microbiol. Immunol..

[22] Sarma J.V., Ward P.A. (2011). The Complement System. Cell Tissue Res..

[23] Qu H., Ricklin D., Lambris J.D. (2009). Recent Developments in Low Molecular Weight Complement Inhibitors. Mol. Immunol..

[24] Klos A., Tenner A.J., Johswich K.O., Ager R.R., Reis E.S., Köhl J. (2009). The role of the anaphylatoxins in health and disease. Mol. Immunol..

[25] Kishore U., Ghai R., Greenhough T.J., Shrive A.K., Bonifati D.M., Gadjeva M.G., Waters P., Kojouharova M.S., Chakraborty T., Agrawal A. (2004). Structural and functional anatomy of the globular domain of complement protein C1q. Immunol. Lett..

[26] Szalai A.J., Agrawal A., Greenhough T.J., Volanakis J.E. (1999). C-Reactive Protein: Structural Biology and Host Defense Function. Clin. Chem. Lab. Med..

[27] Volanakis J.E., Kaplan M.H. (1974). Interaction of C-Reactive Protein Complexes with the Complement system.; I.I. Consumption of Guinea Pig Complement by CRP Complexes: Requirement for Human C1q. J. Immunol..

[28] Kang Y.S., Do Y., Lee H.K., Park S.H., Cheong C., Lynch R.M., Loeffler J.M., Steinman R.M., Park C.G. (2006). A Dominant Complement Fixation Pathway for Pneumococcal Polysaccharides Initiated by SIGN-R1 Interacting with C1q. Cell.

[29] Ebenbichler C.F. (1991). Human immunodeficiency virus type 1 activates the classical pathway of complement by direct C1 binding through specific sites in the transmembrane glycoprotein gp41. J. Exp. Med..

[30] Spear G.T., Jiang H.X., Sullivan B.L., Gewurz H., Landay A.L., Lint T.F. (1991). Direct Binding of Complement Component C1q to Human Immunodeficiency Virus (HIV) and Human T Lymphotrophic Virus-I (HTLV-I) Coinfected Cells. AIDS Res. Hum. Retrovir..

[31] Thielens N.M., Tacnet-Delorme P., Arlaud G.J. (2002). Interaction of C1q and Mannan-binding lectin with viruses. Immunobiology.

[32] Holmskov U., Thiel S., Jensenius J.C. (2003). Collectins and ficolins: Humoral Lectins of the Innate Immune Defense. Annu. Rev. Immunol..

[33] Thiel S., Vorup-Jensen T., Stover C.M., Schwaeble W., Laursen S.B., Poulsen K., Willis A.C., Eggleton P., Hansen S., Holmskov U. (1997). A second serine protease associated with mannan-binding lectin that activates complement. Nature.

[34] Megyeri M., Harmat V., Major B., Végh Á., Balczer J., Héja D., Szilágyi K., Datz D., Pál G., Závodszky P. (2013). Quantitative characterization of the activation steps of mannan-binding lectin (MBL)-associated serine proteases (MASPs) points to the central role of MASP-1 in the initiation of the complement lectin pathway. J. Biol. Chem..

[35] Merle N.S., Church S.E., Fremeaux-Bacchi V., Roumenina L.T. (2015). Complement system part I—Molecular mechanisms of activation and regulation. Front. Immunol..

[36] Pangburn M.K., Schreiber R.D., Müller-Eberhard H.J. (1981). Formation of the initial C3 convertase of the alternative complement pathway. Acquisition of C3b-like activities by spontaneous hydrolysis of the putative thioester in native C3. J. Exp. Med..

[37] Bexborn F., Andersson P.O., Chen H., Nilsson B., Ekdahl K.N. (2008). The Tick-Over Theory Revisited: Formation and Regulation of the soluble Alternative Complement C3 Convertase (C3(H2O)Bb). Mol. Immunol..

[38] Reid K.B.M., Porter R.R. (1981). The Proteolytic Activation Systems of Complement. Annu. Rev. Biochem..

[39] Ganter M.T., Brohi K., Cohen M.J., Shaffer L.A., Walsh M.C., Stahl G.L., Pittet J.F. (2007). Role of the alternative pathway in the early complement activation following major trauma. Shock.

[40] Spitzer D., Mitchell L.M., Atkinson J.P., Hourcade D.E. (2007). Properdin Can Initiate Complement Activation by Binding Specific Target Surfaces and Providing a Platform for De Novo Convertase Assembly. J. Immunol..

[41] Hourcade D.E. (2006). The role of properdin in the assembly of the alternative pathway C3 convertases of complement. J. Biol. Chem..

[42] Harboe M., Mollnes T.E. (2008). The alternative complement pathway revisited. J. Cell Mol. Med..

[43] Walport M.J. (2001). Complement. First of Two Parts. N. Engl. J. Med..

[44] Ehrnthaller C., Ignatius A., Gebhard F., Huber-lang M. (2011). New Insights of an Old Defense System: Structure, Function and Clinical Relevance of the Complement System. Mol. Med..

[45] Koski C.L., Ramm L.E., Hammer C.H., Mayer M.M., Shin M.L. (1983). Cytolysis of nucleated cells by complement: Cell death displays multi-hit characteristics. Proc. Natl. Acad. Sci. USA.

[46] Morgan B.P. (1989). Complement membrane attack on nucleated cells: Resistance, recovery and non-lethal effects. Biochem. J..

[47] Tegla C.A., Cudrici C., Patel S., Trippe R., Rus V., Niculescu F., Rus H. (2011). Membrane attack by complement: The assembly and biology of terminal complement complexes. Immunol. Res..

[48] Kim D.D., Song W. (2006). Membrane complement regulatory proteins. Clin. Immunol..

[49] Schmidt C.Q., Lambris J.D., Ricklin D. (2016). Protection of host cells by complement regulators. Immunol. Rev..

[50] Fearon D.T., Austen K.F. (1975). Properdin: Binding to C3b and stabilization of the C3b dependent C3 convertase. J. Exp. Med..

[51] Fearon D.T. (1979). Regulation of the amplification C3 convertase of human complement by an inhibitory protein isolated from human erythrocyte membrane. Proc. Natl. Acad. Sci. USA.

[52] Fearon D.T. (1980). Identification of the membrane glycoprotein that is the C3b receptor of the human erythrocyte, polymorphonuclear leukocyte, B lymphocyte, and monocyte. J. Exp. Med..

[53] Iida K., Nussenzweig V. (1981). Complement receptor is an inhibitor of the complement cascade. J. Exp. Med..

[54] Medicus R.G., Melamed J., Arnaout M.A. (1983). Role of human factor I and C3b receptor in the cleavage of surface-bound C3bi molecules. Eur. J. Immunol..

[55] Edward Medof M., Iida K., Mold C., Nussenzweig V. (1982). Unique role of the complement receptor CR1 in the degradation of C3b associated with immune complexes. J. Exp. Med..

[56] Seya T., Turner J.R., Atkinson J.P. (1986). Purification and characterization of a membrane protein (gp45-70) that is a cofactor for cleavage of C3B and C4B. J. Exp. Med..

[57] Seya T., Ballard L.L., Bora N.S., Kumar V., Cui W., Atkinson J.P. (1988). Distribution of membrane cofactor protein of complement on human peripheral blood cells. An altered form is found on granulocytes. Eur. J. Immunol..

[58] Liszewski M.K., Post T.W., Atkinson J.P. (1991). Membrane cofactor protein (MCP or CD46): Newest member of the regulators of complement activation gene cluster. Annu. Rev. Immunol..

[59] Nicholson-Weller A., Burge J., Fearon D.T., Weller P.F., Austen K.F. (1982). Isolation of a human erythrocyte membrane glycoprotein with decay-accelerating activity for C3 convertases of the complement system. J. Immunol..

[60] Medof B.Y.M.E., Kinoshita T., Nussenzweig V. (1984). Inhibition of Complement Activation on the Surface of Cells After of Incorporation of Decay-Accelerating Factor (DAF) Into Their Membranes. J. Exp. Med..

[61] Kinoshita T., Medof M.E., Nussenzweig V. (1986). Endogenous association of decay-accelerating factor (DAF) with C4b and C3b on cell membranes. J. Immunol..

[62] Meri S., Morgan B.P., Davies A., Daniels R.H., Olavesen M.G., Waldmann H., Lachmann P.J. (1990). Human protectin (CD59), an 18,000-20,000 MW complement lysis restricting factor, inhibits C5b-8 catalysed insertion of C9 into lipid bilayers. Immunology.

[63] Ninomiya H., Sims P.J. (1992). The human complement regulatory protein CD59 binds to the α-chain of C8 and to the “b” domain of C9. J. Biol. Chem..

[64] Ames R.S., Li Y., Sarau H.M., Nuthulaganti P., Foley J.J., Ellis C., Zeng Z., Su K., Jurewicz A.J., Hertzberg R.P. (1996). Molecular cloning and characterization of the human anaphylatoxin C3a receptor. J. Biol. Chem..

[65] Gerard N.P., Gerard C. (1991). The chemotactic receptor for human C5a anaphylatoxin. Nature.

[66] Ohno M., Hirata T., Enomoto M., Araki T., Ishimaru H., Takahashi T.A. (2000). A putative chemoattractant receptor, C5L2, is expressed in granulocyte and immature dendritic cells, but not in mature dendritic cells. Mol. Immunol..

[67] Okinaga S., Slattery D., Humbles A., Zsengeller Z., Morteau O., Kinrade M.B., Brodbeck R.M., Krause J.E., Choe H.-R., Gerard N.P. (2003). C5L2, a nonsignaling C5A binding protein. Biochemistry.

[68] Van Lith L.H.C., Oosterom J., Van Elsas A., Zaman G.J.R. (2009). C5a-Stimulated Recruitment of ß-Arrestin2 to the Nonsignaling 7-Transmembrane Decoy Receptor C5L2. J. Biomol. Screen..

[69] Croker D.E., Halai R., Kaeslin G., Wende E., Fehlhaber B., Klos A., Monk P.N., Cooper M.A. (2014). C5a2 can modulate ERK1/2 signaling in macrophages via heteromer formation with C5a1 and β-arrestin recruitment. Immunol. Cell Biol..

[70] Hartmann K., Henz B.M., Krüger-Krasagakes S., Köhl J., Burger R., Gurtl S., Haase I., Lippert U., Zuberbier T. (1997). C3a and C5a stimulate chemotaxis of human mast cells. Blood.

[71] Nilsson C., Johnell M., Hammer C.H., Tiffany H.L., Metcalfe D.D., Siegbahn A., Murphyt P.M. (1996). C3a and C5a Are Chemotaxins for Human Mast Cells and Act Through Distinct Receptors via a Pertussis Toxin-Sensitive Signal Transduction Pathway. J. Immunol..

[72] Daffern B.P.J., Pfeifer P.H., Ember J.A., Hugli T.E. (1995). C3a is a Chemotaxin for Human Eosinophils but Not for Neutrophils. I. C3a Stimulation of Neutrophils Is Secondary to Eosinophil Activation. J. Exp. Med..

[73] Aksamit R.R., Falk W., Leonard E.J. (1981). Chemotaxis by mouse macrophage cell lines. J. Immunol..

[74] Yancey K.B., Lawley T.J., Dersookian M., Harvath L. (1989). Analysis of the Interaction of Human C5a and C5a des Arg with Human Monocytes and Neutrophils: Flow Cytometric and Chemotaxis Studies. J. Investig. Dermatol..

[75] Ehrengruber M.U., Geiser T., Deranleau D.A. (1994). Activation of human neutrophils by C3a and C5a. FEBS Lett..

[76] Lett-Brown M.A., Leonard E.J. (1977). Histamine-Induced Inhibition of Normal Human Basophil Chemotaxis to C5a. J. Immunol..

[77] Nataf S., Davoust N., Ames R.S., Barnum S.R. (1999). Human T Cells Express the C5a Receptor and Are Chemoattracted to C5a. J. Immunol..

[78] Ottonello L., Corcione A., Tortolina G., Albesiano E., Favre A., Agostino R.D., Malavasi F., Pistoia V., Dallegri F., Favre A. (1999). rC5a Directs the In Vitro Migration of Human Memory and Naive Tonsillar B Lymphocytes: Implications for B Cell Trafficking in Secondary Lymphoid Tissues. J. Immunol..

[79] Van Lookeren Campagne M., Wiesmann C., Brown E.J. (2007). Macrophage complement receptors and pathogen clearance. Cell. Microbiol..

[80] Karsten C.M., Köhl J. (2012). The immunoglobulin, IgG Fc receptor and complement triangle in autoimmune diseases. Immunobiology.

[81] Fang Y., Xu C., Fu Y.X., Holers V.M., Molina H. (1998). Expression of Complement Receptors 1 and 2 on Follicular Dendritic Cells Is Necessary for the Generation of a Strong Antigen-Specific IgG Response. J. Immunol..

[82] Carter R.H., Fearon D.T. (1992). CD19: Lowering the threshold for antigen receptor stimulation of B lymphocytes. Science.

[83] Cherukuri A., Cheng P.C., Pierce S.K. (2001). The Role of the CD19/CD21 Complex in B Cell Processing and Presentation of Complement-Tagged Antigens. J. Immunol..

[84] Matsumoto A.K., Kopicky-Burd J., Carter R.H., Tuveson D.A., Tedder T.F., Fearon D.T. (1991). Intersection of the Complement and Immune Systems: A Signal Transduction Complex of the B Lymphocyte-containing Complement Receptor Type 2 and CD19. J. Exp. Med..

[85] Strainic M.G., Liu J., Huang D., An F., Lalli P.N., Muqim N., Shapiro V.S., Dubyak G.R., Heeger P.S., Medof M.E. (2008). Locally Produced Complement Fragments C5a and C3a Provide Both Costimulatory and Survival Sinals to naive CD4+ T Cells. Immunity.

[86] Lalli P.N., Strainic M.G., Yang M., Lin F., Medof M.E., Heeger P.S. (2008). Locally produced C5a binds to T cell expressed C5aR to enhance effector T-cell expansion by limiting antigen-induced apoptosis. Blood.

[87] Strainic M.G., Shevach E.M., An F., Lin F., Medof M.E. (2013). Absent C3a and C5a receptor signaling into CD4+T cells enables auto-inductive TGF-β1 signaling and induction of Foxp3+T regulatory cells. Nat. Immunol..

[88] Lalli P.N., Strainic M.G., Lin F., Medof M.E., Heeger P.S. (2007). Decay Accelerating Factor Can Control T Cell Differentiation into IFN-γ-Producing Effector Cells via Regulating Local C5a-Induced IL-12 Production. J. Immunol..

[89] Vadrevu S.K., Chintala N.K., Sharma S.K., Sharma P., Cleveland C., Riediger L., Manne S., Fairlie D.P., Gorczyca W., Almanza O. (2014). Complement C5a receptor facilitates cancer metastasis by altering t-cell responses in the metastatic niche. Cancer Res..

[90] Ytting H., Jensenius J.C., Christensen I.J., Thiel S., Nielsen H.J. (2004). Increased activity of the mannan-binding lectin complement activation pathway in patients with colorectal cancer. Scand. J. Gastroenterol..

[91] Corrales L., Ajona D., Rafail S., Lasarte J.J., Riezu-Boj J.I., Lambris J.D., Rouzaut A., Pajares M.J., Montuenga L.M., Pio R. (2012). Anaphylatoxin C5a creates a favorable microenvironment for lung cancer progression. J. Immunol..

[92] Ajona D., Pajares M.J., Chiara M., Rodrigo J.P., Jantus-Lewintre E., Camps C., Suarez C., Bagán J., Montuenga L., Pio R. (2015). Complement activation product C4d in oral and oropharyngeal squamous cell carcinoma. Oral Dis..

[93] Bu X., Zheng Z., Wang C., Yu Y. (2007). Significance of C4d deposition in the follicular lymphoma and MALT lymphoma and their relationship with follicular dendritic cells. Pathol. Res. Pract..

[94] Bouwens T.A.M., Trouw L.A., Veerhuis R., Dirven C.M.F., Lamfers M.L.M., Al-khawaja H. (2015). Complement activation in Glioblastoma Multiforme pathophysiology: Evidence from serum levels and presence of complement activation products in tumor tissue. J. Neuroimmunol..

[95] Niculescu F., Rus H.G., Retegan M., Vlaicu R. (1992). Persistent Complement Activation on Tumor Cells in Breast Cancer. Am. J. Pathol..

[96] Chen J., Yang W., Sun H., Yang X., Wu Y. (2016). C5b-9 Staining Correlates With Clinical and Tumor Stage in Gastric Adenocarcinoma. Appl. Immunohistochem. Mol. Morphol..

[97] Yamakawa M., Yamada K., Tsuge T., Ohrui H., Ogata T., Dobashi M., Imai Y. (1994). Protection of Thyroid Cancer Cells by Complement-Regulatory Factors. Cancer.

[98] Bjørge L., Hakulinen J., Vintermyr O.K., Jarva H., Jensen T.S., Iversen O.E., Meri S. (2005). Ascitic complement system in ovarian cancer. Br. J. Cancer.

[99] Fishelson Z., Kirschfink M. (2019). Complement C5b-9 and cancer: Mechanisms of cell damage, cancer counteractions, and approaches for intervention. Front. Immunol..

[100] Maestri C.A., Nisihara R., Mendes H.W., Jensenius J., Thiel S., Messias-Reason I., De Carvalho N.S. (2018). MASP-1 and MASP-2 serum levels are associated with worse prognostic in cervical cancer progression. Front. Immunol..

[101] Ytting H., Christensen I.J., Thiel S., Jensenius J.C., Nielsen H.J. (2005). Serum mannan-binding lectin-associated serine protease 2 levels in colorectal cancer: Relation to recurrence and mortality. Clin. Cancer Res..

[102] Swierzko A.S., Szala A., Sawicki S., Szemraj J., Sniadecki M., Sokolowska A., Kaluzynski A., Wydra D., Cedzynski M. (2014). Mannose-Binding Lectin (MBL) and MBL-associated serine protease-2 (MASP-2) in women with malignant and benign ovarian tumours. Cancer Immunol. Immunother..

[103] Fishelson Z., Donin N., Zell S., Schultz S., Kirschfink M. (2003). Obstacles to cancer immunotherapy: Expression of membrane complement regulatory proteins (mCRPs) in tumors. Mol. Immunol..

[104] Olcina M.M., Balanis N.G., Kim R.K., Aksoy B.A., Kodysh J., Thompson M.J., Hammerbacher J., Graeber T.G., Giaccia A.J. (2018). Mutations in an Innate Immunity Pathway Are Associated with Poor Overall Survival Outcomes and Hypoxic Signaling in Cancer. Cell Rep..

[105] Ong H.T., Timm M.M., Greipp P.R., Witzig T.E., Dispenzieri A., Russell S.J., Peng K.W. (2006). Oncolytic measles virus targets high CD46 expression on multiple myeloma cells. Exp. Hematol..

[106] Lok A., Descamps G., Tessoulin B., Chiron D., Eveillard M., Godon C., Le Bris Y., Vabret A., Bellanger C., Maillet L. (2018). P53 regulates CD46 expression and measles virus infection in myeloma cells. Blood Adv..

[107] Watson N.F.S., Durrant L.G., Madjd Z., Ellis I.O., Scholefield J.H., Spendlove I. (2006). Expression of the membrane complement regulatory protein CD59 (protectin) is associated with reduced survival in colorectal cancer patients. Cancer Immunol. Immunother..

[108] Geller A., Yan J. (2019). The Role of Membrane Bound Complement Regulatory Proteins in Tumor Development and Cancer Immunotherapy. Front. Immunol..

[109] Ajona D., Castaño Z., Garayoa M., Zudaire E., Pajares M.J., Martinez A., Cuttitta F., Montuenga L.M., Pio R. (2004). Expression of complement factor H by lung cancer cells: Effects on the activation of the alternative pathway of complement. Cancer Res..

[110] Ajona D., Hsu Y.-F., Corrales L., Montuenga L.M., Pio R. (2007). Down-Regulation of Human Complement Factor H Sensitizes Non-Small Cell Lung Cancer Cells to Complement Attack and Reduces In Vivo Tumor Growth. J. Immunol..

[111] Okroj M., Hsu Y.F., Ajona D., Pio R., Blom A.M. (2008). Non-small cell lung cancer cells produce a functional set of complement factor I and its soluble cofactors. Mol. Immunol..

[112] Laskowski J., Renner B., Pickering M.C., Serkova N.J., Smith-Jones P.M., Clambey E.T., Nemenoff R.A., Thurman J.M. (2020). Complement factor H–deficient mice develop spontaneous hepatic tumors. J. Clin. Investig..

[113] Ajona D., Ortiz-Espinosa S., Pio R., Lecanda F. (2019). Complement in Metastasis: A Comp in the Camp. Front. Immunol..

[114] Whiteside T.L. (2008). The tumor microenvironment and its role in promoting tumor growth. Oncogene.

[115] Cardone J., Friec G.L., Vantourout P., Roberts A., Fuchs A., Jackson I., Suddason T., Lord G., Atkinson J.P., Cope A. (2010). Complement regulator CD46 temporally regulates cytokine production by conventional and unconventional T cells. Nat. Immunol..

[116] Liszewski M.K., Kolev M., Le Friec G., Leung M., Bertram P.G., Fara A.F., Subias M., Pickering M.C., Drouet C., Meri S. (2013). Intracellular Complement Activation Sustains T Cell Homeostasis and Mediates Effector Differentiation. Immunity.

[117] Kolev M., Dimeloe S., Le Friec G., Navarini A., Arbore G., Povoleri G.A., Fischer M., Belle R., Loeliger J., Develioglu L. (2015). Complement Regulates Nutrient Influx and Metabolic Reprogramming during Th1 Cell Responses. Immunity.

[118] Cho M.S., Vasquez H.G., Rupaimoole R., Pradeep S., Wu S., Zand B., Han H.D., Rodriguez-Aguayo C., Bottsford-Miller J., Huang J. (2014). Autocrine Effects of Tumor-Derived Complement. Cell Rep..

[119] Bulla R., Tripodo C., Rami D., Ling G.S., Agostinis C., Guarnotta C., Zorzet S., Durigutto P., Botto M., Tedesco F. (2016). C1q acts in the tumour microenvironment as a cancer-promoting factor independently of complement activation. Nat. Commun..

[120] Riihilä P., Nissinen L., Farshchian M., Kivisaari A., Ala-aho R., Kallajoki M., Grénman R., Meri S., Peltonen S., Peltonen J. (2015). Complement factor I promotes progression of cutaneous squamous cell carcinoma. J. Investig. Dermatol..

[121] Riihilä P., Nissinen L., Farshchian M., Kallajoki M., Kivisaari A., Meri S., Grénman R., Peltonen S., Peltonen J., Pihlajaniemi T. (2017). Complement Component C3 and Complement Factor B Promote Growth of Cutaneous Squamous Cell Carcinoma. Am. J. Pathol..

[122] Fan Z., Qin J., Wang D., Geng S. (2019). Complement C3a promotes proliferation, migration and stemness in cutaneous squamous cell carcinoma. J. Cell. Mol. Med..

[123] Nunez-Cruz S., Gimotty P.A., Guerra M.W., Connolly D.C., Wu Y.Q., DeAngelis R.A., Lambris J.D., Coukos G., Scholler N. (2012). Genetic and pharmacologic inhibition of complement impairs endothelial cell function and ablates ovarian cancer neovascularization. Neoplasia.

[124] Yoneda M., Imamura R., Nitta H., Taniguchi K., Saito F., Kikuchi K., Ogi H., Tanaka T., Katabuchi H., Nakayama H. (2019). Enhancement of cancer invasion and growth via the C5a-C5a receptor system: Implications for cancer promotion by autoimmune diseases and association with cervical cancer invasion. Oncol. Lett..

[125] Maeda Y., Kawano Y., Wada Y., Yatsuda J., Motoshima T., Murakami Y., Kikuchi K., Imamura T., Eto M. (2015). C5aR is frequently expressed in metastatic renal cell carcinoma and plays a crucial role in cell invasion via the ERK and PI3 kinase pathways. Oncol. Rep..

[126] Hu W.H., Hu Z., Shen X., Dong L.-Y., Zhou W.-Z., Yu X.-X. (2016). C5a receptor enhances hepatocellular carcinoma cell invasiveness via activating ERK1/2-mediated epithelial-mesenchymal transition. Exp. Mol. Pathol..

[127] Ajona D., Zandueta C., Corrales L., Moreno H., Pajares M.J., Ortiz-Espinosa S., Martínez-Terroba E., Perurena N., De Miguel F.J., Jantus-Lewintre E. (2018). Blockade of the complement C5a/C5aR1 axis impairs lung cancer bone metastasis by CXCL16-mediated effects. Am. J. Respir. Crit. Care Med..

[128] Abdelbaset-Ismail A., Borkowska-Rzeszotek S., Kubis E., Bujko K., Brzeźniakiewicz-Janus K., Bolkun L., Kloczko J., Moniuszko M., Basak G.W., Wiktor-Jedrzejczak W. (2017). Activation of the complement cascade enhances motility of leukemic cells by downregulating expression of HO-1. Leukemia.

[129] Nitta H., Wada Y., Kawano Y., Murakami Y., Irie A., Taniguchi K., Kikuchi K., Yamada G., Suzuki K., Honda J. (2004). Enhancement of Human Cancer Cell Motility and Invasiveness by Anaphylatoxin C5a via Aberrantly Expressed C5a Receptor ( CD88 ). Clin. Cancer Res..

[130] Cho M.S., Rupaimoole R., Choi H.-J., Noh K., Chen J., Hu Q., Sood A.K., Afshar-Kharghan V. (2016). Complement Component 3 Is Regulated by TWIST1 and Mediates Epithelial—Mesenchymal Transition. J. Immunol..

[131] Kochanek D.M., Ghouse S.M., Karbowniczek M.M., Markiewski M.M. (2018). Complementing cancer metastasis. Front. Immunol..

[132] Block I., Müller C., Sdogati D., Pedersen H., List M., Jaskot A.M., Syse S.D., Lund Hansen P., Schmidt S., Christiansen H. (2019). CFP suppresses breast cancer cell growth by TES-mediated upregulation of the transcription factor DDIT3. Oncogene.

[133] Bandini S., Curcio C., Macagno M., Quaglino E., Arigoni M., Lanzardo S., Hysi A., Barutello G., Consolino L., Longo D.L. (2013). Early onset and enhanced growth of autochthonous mammary carcinomas in C3-deficient Her2/neu transgenic mice. Oncoimmunology.

[134] Bandini S., Macagno M., Hysi A., Lanzardo S., Conti L., Bello A., Riccardo F., Ruiu R., Merighi I.F., Forni G. (2016). The non-inflammatory role of C1q during Her2/neu-driven mammary carcinogenesis. Oncoimmunology.

[135] Kim D.Y., Martin C.B., Soon N.L., Martin B.K. (2005). Expression of complement protein C5a in a murine mammary cancer model: Tumor regression by interference with the cell cycle. Cancer Immunol. Immunother..

[136] Wang Y., Sun S.-N., Liu Q., Yu Y., Guo J., Wang K., Xing B.-C., Zheng Q.-F., Campa M.J., Patz E.F. (2016). Autocrine complement inhibits IL10-dependent T-cell-mediated antitumor immunity to promote tumor progression. Cancer Discov..

[137] Ajona D., Ortiz-Espinosa S., Moreno H., Lozano T., Pajares M.J., Agorreta J., Bértolo C., Lasarte J.J., Vicent S., Hoehlig K. (2017). A combined PD-1/C5a blockade synergistically protects against lung cancer growth and metastasis. Cancer Discov..

[138] Downs-Canner S., Magge D., Ravindranathan R., O’Malley M.E., Francis L., Liu Z., Sheng Guo Z., Obermajer N., Bartlett D.L. (2016). Complement Inhibition: A Novel Form of Immunotherapy for Colon Cancer. Ann. Surg. Oncol..

[139] Ding P., Li L., Li L., Lv X., Zhou D., Wang Q., Chen J., Yang C., Xu E., Dai W. (2020). C5aR1 is a master regulator in colorectal tumorigenesis via immune modulation. Theranostics.

[140] Gunn L., Ding C., Liu M., Ma Y., Qi C., Cai Y., Hu X., Aggarwal D., Zhang H., Yan J. (2012). Opposing Roles for Complement Component C5a in Tumor Progression and the Tumor Microenvironment. J. Immunol..

[141] Kwak J.W., Laskowski J., Li H.Y., McSharry M.V., Sippel T.R., Bullock B.L., Johnson A.M., Poczobutt J.M., Neuwelt A.J., Malkoski S.P. (2018). Complement activation via a C3a receptor pathway alters CD4+ T lymphocytes and mediates lung cancer progression. Cancer Res..

[142] Xu Y., Huang Y., Xu W., Zheng X., Yi X., Huang L., Wang Y., Wu K. (2020). Activated hepatic stellate cells (HSCs) exert immunosuppressive effects in hepatocellular carcinoma by producing complement C3. Onco. Targets. Ther..

[143] Guglietta S., Chiavelli A., Zagato E., Krieg C., Gandini S., Ravenda P.S., Bazolli B., Lu B., Penna G., Rescigno M. (2016). Coagulation induced by C3aR-dependent NETosis drives protumorigenic neutrophils during small intestinal tumorigenesis. Nat. Commun..

[144] Ning C., Li Y.-Y., Wang Y., Han G.C., Wang R.X., Xiao H., Li X.Y., Hou C.M., Ma Y.F., Sheng D.S. (2015). Complement activation promotes colitis-associated carcinogenesis through activating intestinal IL-1β/IL-17A axis. Mucosal Immunol..

[145] Nabizadeh J.A., Manthey H.D., Steyn F.J., Chen W., Widiapradja A., Md Akhir F.N., Boyle G.M., Taylor S.M., Woodruff T.M., Rolfe B.E. (2016). The Complement C3a Receptor Contributes to Melanoma Tumorigenesis by Inhibiting Neutrophil and CD4 + T Cell Responses. J. Immunol..

[146] Davidson S., Efremova M., Riedel A., Mahata B., Pramanik J., Huuhtanen J., Kar G., Vento-Tormo R., Hagai T., Chen X. (2020). Single-Cell RNA Sequencing Reveals a Dynamic Stromal Niche That Supports Tumor Growth. Cell Rep..

[147] Bonavita E., Gentile S., Rubino M., Maina V., Papait R., Kunderfranco P., Greco C., Feruglio F., Molgora M., Laface I. (2015). PTX3 is an extrinsic oncosuppressor regulating complement-dependent inflammation in cancer. Cell.

[148] Piao C., Cai L., Qiu S., Jia L., Song W., Du J. (2015). Complement 5a enhances hepatic metastases of colon cancer via monocyte chemoattractant protein-1-mediated inflammatory cell infiltration. J. Biol. Chem..

[149] Contractor T., Kobayashi S., da Silva E., Clausen R., Chan C., Vosburgh E., Tang L.H., Levine A.J., Harris C.R. (2016). Sexual dimorphism of liver metastasis by murine pancreatic neuroendocrine tumors is affected by expression of complement C5. Oncotarget.

[150] Piao C., Zhang W.M., Li T.T., Zhang C.C., Qiu S., Liu Y., Liu S., Jin M., Jia L.X., Song W.C. (2018). Complement 5a stimulates macrophage polarization and contributes to tumor metastases of colon cancer. Exp. Cell Res..

[151] Medler T.R., Murugan D., Horton W., Kumar S., Cotechini T., Forsyth A.M., Leyshock P., Leitenberger J.J., Kulesz-Martin M., Margolin A.A. (2018). Complement C5a Fosters Squamous Carcinogenesis and Limits T Cell Response to Chemotherapy. Cancer Cell.

[152] Janelle V., Langlois M.P., Tarrab E., Lapierre P., Poliquin L., Lamarre A. (2014). Transient complement inhibition promotes a tumor-specific immune response through the implication of natural killer cells. Cancer Immunol. Res..

[153] Liu C.-F., Min X.-Y., Wang N., Wang J.-X., Ma N., Dong X., Zhang B., Wu W., Li Z.F., Zhou W. (2017). Complement receptor 3 has negative impact on tumor surveillance through suppression of natural killer cell function. Front. Immunol..

[154] Gu J., Ding J.Y., Lu C.L., Lin Z.W., Chu Y.W., Zhao G.Y., Guo J., Ge D. (2013). Overexpression of CD88 predicts poor prognosis in non-small-cell lung cancer. Lung Cancer.

[155] Yuan K., Ye J., Liu Z., Ren Y., He W., Xu J., He Y., Yuan Y. (2020). Complement C3 overexpression activates JAK2/STAT3 pathway and correlates with gastric cancer progression. J. Exp. Clin. Cancer Res..

[156] Chen J., Li G.Q., Zhang L., Tang M., Cao X., Xu G.L., Wu Y.Z. (2018). Complement C5a/C5aR pathway potentiates the pathogenesis of gastric cancer by down-regulating p21 expression. Cancer Lett..

[157] Lin K., He S., He L., Chen J., Cheng X., Zhang G., Zhu B. (2014). Complement component 3 is a prognostic factor of non-small cell lung cancer. Mol. Med. Rep..

[158] Baskar R., Lee K.A., Yeo R., Yeoh K.W. (2012). Cancer and radiation therapy: Current advances and future directions. Int. J. Med. Sci..

[159] Borras J.M., Lievens Y., Barton M., Corral J., Ferlay J., Bray F., Grau C. (2016). How many new cancer patients in Europe will require radiotherapy by 2025? An ESTRO-HERO analysis. Radiother. Oncol..

[160] Surace L., Lysenko V., Fontana A.O., Cecconi V., Janssen H., Bicvic A., Okoniewski M., Pruschy M., Dummer R., Neefjes J. (2015). Complement Is a Central Mediator of Radiotherapy-Induced Tumor-Specific Immunity and Clinical Response. Immunity.

[161] Demaria S., Formenti S.C. (2012). Role of T lymphocytes in tumor response to radiotherapy. Front. Oncol..

[162] Eriksson D., Stigbrand T. (2010). Radiation-induced cell death mechanisms. Tumor Biol..

[163] Apetoh L., Ghiringhelli F., Tesniere A., Obeid M., Ortiz C., Criollo A., Mignot G., Maiuri M.C., Ullrich E., Saulnier P. (2007). Toll-like receptor 4-dependent contribution of the immune system to anticancer chemotherapy and radiotherapy. Nat. Med..

[164] Matsumura S., Wang B., Kawashima N., Braunstein S., Badura M., Cameron T.O., Babb J.S., Schneider R.J., Formenti S.C., Dustin M.L. (2008). Radiation-induced CXCL16 release by breast cancer cells attracts effector T cells. J. Immunol..

[165] Reits E.A., Hodge J.W., Herberts C.A., Groothuis T.A., Chakraborty M., Wansley E.K., Camphausen K., Luiten R.M., De Ru A.H., Neijssen J. (2006). Radiation modulates the peptide repertoire, enhances MHC class I expression, and induces successful antitumor immunotherapy. J. Exp. Med..

[166] Song H., He C., Knaak C., Guthridge J.M., Holers V.M., Tomlinson S. (2003). Complement receptor 2-mediated targeting of complement inhibitors to sites of complement activation. J. Clin. Investig..

[167] Engelman R.M., Rousou J.A., Flack J.E., Deaton D.W., Kalfin R., Das D.K. (1995). Influence of steroids on complement and cytokine generation after cardiopulmonary bypass. Ann. Thorac. Surg..

[168] Coulpier M., Andreev S., Lemercier C., Dauchel H., Lees O., Fontaine M., Ripoche J. (1995). Activation of the endothelium by IL-la and glucocorticoids results in major increase of complement C3 and factor B production and generation of C3a. Clin. Exp. Immunol..

[169] Lappin D.F., Whaley K. (1991). Modulation of complement gene expression by glucocorticoids. Biochem. J..

[170] Schleimer R.P. (2004). Glucocorticoids suppress inflammation but spare innate immune responses in airway epithelium. Proc. Am. Thorac. Soc..

[171] Burnette B.C., Liang H., Lee Y., Chlewicki L., Khodarev N.N., Weichselbaum R.R., Fu Y.X., Auh S.L. (2011). The efficacy of radiotherapy relies upon induction of type I interferon-dependent innate and adaptive immunity. Cancer Res..

[172] Lee Y., Auh S.L., Wang Y., Burnette B., Wang Y., Meng Y., Beckett M., Sharma R., Chin R., Tu T. (2009). Therapeutic effects of ablative radiation on local tumor require CD8 + T cells: Changing strategies for cancer treatment. Blood.

[173] Dewan M.Z., Galloway A.E., Kawashima N., Dewyngaert J.K., Babb J.S., Formenti S.C., Demaria S. (2009). Fractionated but not single-dose radiotherapy induces an immune-mediated abscopal effect when combined with anti-CTLA-4 antibody. Clin. Cancer Res..

[174] Dovedi S.J., Illidge T.M. (2015). The antitumor immune response generated by fractionated radiation therapy may be limited by tumor cell adaptive resistance and can be circumvented by PD-L1 blockade. Oncoimmunology.

[175] Dovedi S.J., Melis M.H.M., Wilkinson R.W., Adlard A.L., Stratford I.J., Honeychurch J., Illidge T.M. (2013). Systemic delivery of a TLR7 agonist in combination with radiation primes durable antitumor immune responses in mouse models of lymphoma. Blood.

[176] Lu Y., Zhao Q., Liao J.Y., Song E., Xia Q., Pan J., Li Y., Li J., Zhou B., Ye Y. (2020). Complement Signals Determine Opposite Effects of B Cells in Chemotherapy-Induced Immunity. Cell.

[177] Sautès-Fridman C., Roumenina L.T. (2020). B cells and complement at the forefront of chemotherapy. Nat. Rev. Clin. Oncol..

[178] Murray K.P., Mathure S., Kaul R., Khan S., Carson L.F., Twiggs L.B., Martens M.G., Kaul A. (2000). Expression of complement regulatory proteins - CD 35, CD 46, CD 55, and CD 59 - In benign and malignant endometrial tissue. Gynecol. Oncol..

[179] Saygin C., Wiechert A., Rao V.S., Alluri R., Connor E., Thiagarajan P.S., Hale J.S., Li Y., Chumakova A., Jarrar A. (2017). CD55 regulates self-renewal and cisplatin resistance in endometrioid tumors. J. Exp. Med..

[180] Hammond E.M., Asselin M.C., Forster D., O’Connor J.P.B., Senra J.M., Williams K.J. (2014). The Meaning, Measurement and Modification of Hypoxia in the Laboratory and the Clinic. Clin. Oncol..

[181] Muz B., de la Puente P., Azab F., Azab A.K. (2015). The role of hypoxia in cancer progression, angiogenesis, metastasis, and resistance to therapy. Hypoxia.

[182] Rankin E.B., Giaccia A.J. (2016). Hypoxic control of metastasis. Science.

[183] Gray L.H., Conger A.D., Ebert M., Hornsey S., Scott O.C. (1953). The concentration of oxygen dissolved in tissues at the time of irradiation as a factor in radiotherapy. Br. J. Radiol..

[184] Riley P.A. (1994). Free radicals in biology: Oxidative stress and the effects of ionizing radiation. Int. J. Radiat. Biol..

[185] Graham K., Unger E. (2018). Overcoming tumor hypoxia as a barrier to radiotherapy, chemotherapy and immunotherapy in cancer treatment. Int. J. Nanomedicine.

[186] Nordsmark M., Bentzen S.M., Rudat V., Brizel D., Lartigau E., Stadler P., Becker A., Adam M., Molls M., Dunst J. (2005). Prognostic value of tumor oxygenation in 397 head and neck tumors after primary radiation therapy. An international multi-center study. Radiother. Oncol..

[187] Brizel D.M., Dodge R.K., Clough R.W., Dewhirst M.W. (1999). Oxygenation of head and neck cancer: Changes during radiotherapy and impact on treatment outcome. Radiother. Oncol..

[188] Höckel M., Schlenger K., Aral B., Mitze M., Schäffer U., Vaupel P. (1996). Association between tumor hypoxia and malignant progression in advanced cancer of the uterine cervix. Cancer Res..

[189] Koch S., Mayer F., Honecker F., Schittenhelm M., Bokemeyer C. (2003). Efficacy of cytotoxic agents used in the treatment of testicular germ cell tumours under normoxic and hypoxic conditions in vitro. Br. J. Cancer.

[190] Strese S., Fryknäs M., Larsson R., Gullbo J. (2013). Effects of hypoxia on human cancer cell line chemosensitivity. BMC Cancer.

[191] Okroj M., Corrales L., Stokowska A., Pio R., Blom A.M. (2009). Hypoxia increases susceptibility of non-small cell lung cancer cells to complement attack. Cancer Immunol. Immunother..

[192] Wenger R.H., Rolfs A., Marti H.H., Bauer C., Gassmann M. (1995). Hypoxia, a novel inducer of acute phase gene expression in a human hepatoma cell line. J. Biol. Chem..

[193] Louis N.A., Hamilton K.E., Kong T., Colgan S.P. (2005). HIF-dependent induction of apical CD55 coordinates epithelial clearance of neutrophils. FASEB J..

[194] Olcina M.M., Kim R.K., Melemenidis S., Graves E.E., Giaccia A.J. (2019). The tumour microenvironment links complement system dysregulation and hypoxic signalling. Br. J. Radiol..

[195] Jackson S.P., Bartek J. (2009). The DNA-damage response in human biology and disease. Nature.

[196] O’Connor M.J. (2015). Targeting the DNA Damage Response in Cancer. Mol. Cell.

[197] Lord C.J., Ashworth A. (2012). The DNA damage response and cancer therapy. Nature.

[198] Lynam-Lennon N., Reynolds J.V., Pidgeon G.P., Lysaght J., Marignol L., Maher S.G. (2010). Alterations in DNA Repair Efficiency are Involved in the Radioresistance of Esophageal Adenocarcinoma. Radiat. Res..

[199] Bao S., Wu Q., McLendon R.E., Hao Y., Shi Q., Hjelmeland A.B., Dewhirst M.W., Bigner D.D., Rich J.N. (2006). Glioma stem cells promote radioresistance by preferential activation of the DNA damage response. Nature.

[200] Rocha C.R.R., Silva M.M., Quinet A., Cabral-Neto J.B., Menck C.F.M. (2018). DNA repair pathways and cisplatin resistance: An intimate relationship. Clinics.

[201] Lynam-Lennon N., Bibby B.A.S., Mongan A.M., Marignol L., Paxton C.N., Geiersbach K., Bronner M.P., Sullivan J.O., Reynolds J.V., Maher S.G. (2016). Low MiR-187 Expression Promotes Resistance to Chemoradiation Therapy In Vitro and Correlates with Treatment Failure in Patients with Esophageal Adenocarcinoma. Mol. Med..

[202] Kurumizaka H., Ikawa S., Nakada M., Eda K., Kagawa W., Takata M., Takeda S., Yokoyama S., Shibata T. (2001). Homologous-pairing activity of the human DNA-repair proteins Xrcc3.Rad51C. Proc. Natl. Acad. Sci. USA.

[203] Pei J.S., Chang W.S., Hsu P.C., Chen C.C., Cheng S.P., Wang Y.C., Tsai C.W., Shen T.C., Bau D.T. (2018). The contribution of XRCC3 genotypes to childhood acute lymphoblastic leukemia. Cancer Manag. Res..

[204] Kuricova M., Naccarati A., Kumar R., Koskinen M., Sanyal S., Dusinska M., Tulinska J., Vodickova L., Liskova A., Jahnova E. (2005). DNA repair and cyclin D1 polymorphisms and styrene-induced genotoxicity and immunotoxicity. Toxicol. Appl. Pharmacol..

[205] Warburg O. (1956). On the origin of cancer. Science.

[206] Heiden M.G.V., Cantley L.C., Thompson C.B. (2009). Understanding the warburg effect: The metabolic requirements of cell proliferation. Science.

[207] Rashmi R., Huang X., Floberg J.M., Elhammali A.E., McCormick M.L., Patti G.J., Spitz D.R., Schwarz J.K. (2018). Radio-resistant cervical cancers are sensitive to inhibition of glycolysis and redox metabolism. Cancer Res..

[208] Chakraborty P.K., Mustafi S.B., Xiong X., Dwivedi S.K.D., Nesin V., Saha S., Zhang M., Dhanasekaran D., Jayaraman M., Mannel R. (2017). MICU1 drives glycolysis and chemoresistance in ovarian cancer. Nat. Commun..

[209] Cascone T., McKenzie J.A., Mbofung R.M., Punt S., Wang Z., Xu C., Williams L.J., Wang Z., Bristow C.A., Carugo A. (2018). Increased Tumor Glycolysis Characterizes Immune Resistance to Adoptive T Cell Therapy. Cell Metab..

[210] Ruprecht B., Zaal E.A., Zecha J., Wu W., Berkers C.R., Kuster B., Lemeer S. (2017). Lapatinib resistance in breast cancer cells is accompanied by phosphorylation-mediated reprogramming of glycolysis. Cancer Res..

[211] Bhattacharya B., Low S.H.H., Soh C., Kamal Mustapa N., Beloueche-Babari M., Koh K.X., Loh J., Soong R. (2014). Increased drug resistance is associated with reduced glucose levels and an enhanced glycolysis phenotype. Br. J. Pharmacol..

[212] Hess C., Kemper C. (2016). Complement-Mediated Regulation of Metabolism and Basic Cellular Processes. Immunity.

[213] Kolev M., Kemper C. (2017). Keeping it all going-complement meets metabolism. Front. Immunol..

[214] West E.E., Kemper C. (2019). Complement and T Cell Metabolism: Food for Thought. Immunometabolism.

[215] Arbore G., West E.E., Spolski R., Robertson A.A.B., Klos A., Rheinheimer C., Dutow P., Woodruff T.M., Yu Z.X., O’Neill L.A. (2016). T helper 1 immunity requires complement-driven NLRP3 inflammasome actvity in CD4+ T cells. Science.

[216] Jiang Y., Li Y., Zhu B. (2015). T-cell exhaustion in the tumor microenvironment. Cell Death Dis..

[217] Manning B.D., Cantley L.C. (2007). AKT/PKB Signaling: Navigating Downstream. Cell.

[218] Fresno Vara J.Á., Casado E., de Castro J., Cejas P., Belda-Iniesta C., González-Barón M. (2004). P13K/Akt signalling pathway and cancer. Cancer Treat. Rev..

[219] Rus H.G., Niculescu F.I., Shin M.L. (2001). Role of the C5b-9 complement complex in cell cycle and apoptosis. Immunol. Rev..

[220] Raposo G., Stoorvogel W. (2013). Extracellular vesicles: Exosomes, microvesicles, and friends. J. Cell Biol..

[221] Colombo M., Raposo G., Théry C. (2014). Biogenesis, secretion, and intercellular interactions of exosomes and other extracellular vesicles. Annu. Rev. Cell Dev. Biol..

[222] Denzer K., Kleijmeer M.J., Heijnen H.F.G., Stoorvogel W., Geuze H.J. (2000). Exosome: From internal vesicle of the multivesicular body to intercellular signaling device. J. Cell Sci..

[223] Blanchard N., Lankar D., Faure F., Regnault A., Dumont C., Raposo G., Hivroz C. (2002). TCR Activation of Human T Cells Induces the Production of Exosomes Bearing the TCR/CD3/ζ Complex. J. Immunol..

[224] Melo S.A., Sugimoto H., Connell J.T.O., Kato N., Vidal A., Qiu L., Vitkin E., Perelman L.T., Melo C.A., Lucci A. (2014). Cancer Exosomes Perform Cell-Independent MicroRNA Biogenesis and Promote Tumorigenesis. Cancer Cell.

[225] Gallo A., Tandon M., Alevizos I., Illei G.G. (2012). The majority of microRNAs detectable in serum and saliva is concentrated in exosomes. PLoS ONE.

[226] Street J.M., Barran P.E., Mackay C.L., Weidt S., Balmforth C., Walsh T.S., Chalmers R.T.A., Webb D.J., Dear J.W. (2012). Identification and proteomic profiling of exosomes in human cerebrospinal fluid. J. Transl. Med..

[227] Van den Boorn J.G., Daßler J., Coch C., Schlee M., Hartmann G. (2013). Exosomes as nucleic acid nanocarriers. Adv. Drug Deliv. Rev..

[228] Barros F.M., Carneiro F., Machado J.C., Melo S.A. (2018). Exosomes and Immune Response in cancer: Friends or Foes?. Front. Immunol..

[229] Corrado C., Raimondo S., Chiesi A., Ciccia F., De Leo G., Alessandro R. (2013). Exosomes as intercellular signaling organelles involved in health and disease: Basic science and clinical applications. Int. J. Mol. Sci..

[230] Azmi A.S., Bao B., Sarkar F.H. (2013). Exosomes in cancer development, metastasis, and drug resistance: A comprehensive review. Cancer Metastasis Rev..

[231] Kalluri R. (2016). The biology and function of exosomes in cancer. J. Clin. Investig..

[232] Al-Nedawi K., Meehan B., Micallef J., Lhotak V., May L., Guha A., Rak J. (2008). Intercellular transfer of the oncogenic receptor EGFRvIII by microvesicles derived from tumour cells. Nat. Cell Biol..

[233] Andreola G., Rivoltini L., Castelli C., Huber V., Perego P., Deho P., Squarcina P., Accornero P., Lozupone F., Lugini L. (2002). Induction of lymphocyte apoptosis by tumor cell secretion of FasL-bearing microvesicles. J. Exp. Med..

[234] Wieckowski E.U., Visus C., Szajnik M., Szczepanski M.J., Storkus W.J., Whiteside T.L. (2009). Tumor-Derived Microvesicles Promote Regulatory T Cell Expansion and Induce Apoptosis in Tumor-Reactive Activated CD8+ T Lymphocytes. J. Immunol..

[235] Peinado H., Alečković M., Lavotshkin S., Matei I., Costa-Silva B., Moreno-Bueno G., Hergueta-Redondo M., Williams C., García-Santos G., Ghajar C.M. (2012). Melanoma exosomes educate bone marrow progenitor cells toward a pro-metastatic phenotype through MET. Nat. Med..

[236] Costa-Silva B., Aiello N.M., Ocean A.J., Singh S., Zhang H., Thakur B.K., Becker A., Hoshino A., Mark M.T., Molina H. (2015). Pancreatic cancer exosomes initiate pre-metastatic niche formation in the liver. Nat. Cell Biol..

[237] Karasu E., Eisenhardt S.U., Harant J., Huber-Lang M. (2018). Extracellular vesicles: Packages sent with complement. Front. Immunol..

[238] Papp K., Végh P., Prechl J., Kerekes K., Kovács J., Csikós G., Bajtay Z., Erdei A. (2008). B lymphocytes and macrophages release cell membrane deposited C3-fragments on exosomes with T cell response-enhancing capacity. Mol. Immunol..

[239] Clayton A., Harris C.L., Court J., Mason M.D., Morgan B.P. (2003). Antigen-presenting cell exosomes are protected from complement-mediated lysis by expression of CD55 and CD59. Eur. J. Immunol..

[240] Chen Y., Xie Y., Xu L., Zhan S., Xiao Y., Gao Y., Wu B., Ge W. (2017). Protein content and functional characteristics of serum-purified exosomes from patients with colorectal cancer revealed by quantitative proteomics. Int. J. Cancer.

[241] Balbinotti H., Cadore N.A., Dutra C.S., Silva E.D.D.A., Ferreira H.B., Zaha A., Monteiro K.M. (2020). Protein profiling of extracellular vesicles associated with cisplatin resistance in lung cancer. Anticancer Res..

[242] Pilzer D., Gasser O., Moskovich O., Schifferli J.A., Fishelson Z. (2005). Emission of membrane vesicles: Roles in complement resistance, immunity and cancer. Springer Semin. Immunopathol..

[243] Pilzer D., Fishelson Z. (2005). Mortalin/GRP75 promotes release of membrane vesicles from immune attacked cells and protection from complement-mediated lysis. Int. Immunol..

[244] Aung T., Chapuy B., Vogel D., Wenzel D., Oppermann M., Lahmann M., Weinhage T., Menck K., Hupfeld T., Koch R. (2011). Exosomal evasion of humoral immunotherapy in aggressive B-cell lymphoma modulated by ATP-binding cassette transporter A3. Proc. Natl. Acad. Sci. USA.

[245] Safaei R., Larson B.J., Cheng T.C., Gibson M.A., Otani S., Naerdemann W., Howell S.B. (2005). Abnormal lysosomal trafficking and enhanced exosomal export of cisplatin in drug-resistant human ovarian carcinoma cells. Mol. Cancer Ther..

[246] Kelleher R., Balu-Iyer S., Loyall J., Sacca A.J., Shenoy G.N., Peng P., Iyer V., Fathallah A.M., Berenson C.S., Wallace P.K. (2015). Extracellular Vesicles Present in Human Ovarian Tumor Microenvironments Induce a Phosphatidylserine-Dependent Arrest in the T-cell Signaling Cascade. Cancer Immunol. Res..

[247] Unnewehr H., Rittirsch D., Sarma J.V., Zetoune F., Flierl M.A., Perl M., Denk S., Weiss M., Schneider M.E., Monk P.N. (2013). Changes and Regulation of the C5a Receptor on Neutrophils during Septic Shock in Humans. J. Immunol..

[248] Reis E.S., Mastellos D.C., Hajishengallis G., Lambris J.D. (2019). New insights into the immune functions of complement. Nat. Rev. Immunol..

[249] Zhang J., Chen M., Zhao Y., Xiong H., Sneh T., Fan Y., Wang J., Zhou X., Gong C. (2020). Complement and coagulation cascades pathway correlates with chemosensitivity and overall survival in patients with soft tissue sarcoma. Eur. J. Pharmacol..

[250] Michlmayr A., Bachleitner-Hofmann T., Baumann S., Marchetti-Deschmann M., Rech-Weichselbraun I., Burghuber C., Pluschnig U., Bartsch R., Graf A., Greil R. (2010). Modulation of plasma complement by the initial dose of epirubicin/docetaxel therapy in breast cancer and its predictive value. Br. J. Cancer.

[251] Maher S.G., McDowell D.T., Collins B.C., Muldoon C., Gallagher W.M., Reynolds J.V. (2011). Serum Proteomic Profiling Reveals That Pretreatment Complement Protein Levels are Predictive of Esophageal Cancer Patient Response to Neoadjuvant Chemoradiation. Ann. Surg..

[252] Farkona S., Diamandis E.P., Blasutig I.M. (2016). Cancer immunotherapy: The beginning of the end of cancer?. BMC Med..

[253] Mellman I., Coukos G., Dranoff G. (2011). Cancer immunotherapy comes of age. Nature.

[254] Dougan M., Dranoff G. (2009). Immune Therapy for Cancer. Annu. Rev. Immunol..

[255] Maloney D.G., Grillo-López A.J., White C.A., Bodkin D., Schilder R.J., Neidhart J.A., Janakiraman N., Foon K.A., Liles T.-M., Dallaire B.K. (1997). IDEC-C2B8 (rituximab) anti-CD20 monoclonal antibody therapy in patients with relapsed low-grade non-Hodgkin’s lymphoma. Blood.

[256] Piccart-Gebhart M.J., Procter M., Leyland-Jones B., Goldhirsch A., Untch M., Smith I., Gianni L., Baselga J., Bell R., Jackisch C. (2005). Trastuzumab after Adjuvant Chemotherapy in HER2-Positive Breast Cancer. N. Engl. J. Med..

[257] De Weers M., Tai Y.-T., van der Veer M.S., Bakker J.M., Vink T., Jacobs D.C.H., Oomen L.A., Peipp M., Valerius T., Slootstra J.W. (2011). Daratumumab, a Novel Therapeutic Human CD38 Monoclonal Antibody, Induces Killing of Multiple Myeloma and Other Hematological Tumors. J. Immunol..

[258] Taylor R.P., Lindorfer M.A. (2016). Cytotoxic mechanisms of immunotherapy: Harnessing complement in the action of anti-tumor monoclonal antibodies. Semin. Immunol..

[259] Carter P., Presta L., Gorman C.M., Ridgway J.B.B., Henner D., Wong W.L.T., Rowland A.M., Kotts C., Carver M.E., Shepard H.M. (1992). Humanization of an anti-p185HER2 antibody for human cancer therapy. Proc. Natl. Acad. Sci. USA.

[260] Clynes R.A., Towers T.L., Presta L.G., Ravetch J.V. (2000). Inhibitory Fc receptors modulate in vivo cytoxicity against tumor targets. Nat. Med..

[261] Taylor R.P., Lindorfer M.A. (2014). The role of complement in mAb-based therapies of cancer. Methods.

[262] Derer S., Beurskens F.J., Rösner T., Peipp M., Valerius T. (2014). Complement in antibody-based tumor therapy. Crit. Rev. Immunol..

[263] Golay J., Zaffaroni L., Vaccari T., Lazzari M., Borleri G.-M., Bernasconi S., Tedesco F., Rambaldi A., Introna M. (2000). Biologic response of Blymphoma cells to anti-CD20 monoclonal antibody rituximab in vitro: CD55 and CD59 regulate complement-mediated cell lysis. Blood.

[264] Rogers L.M., Veeramani S., Weiner G.J. (2014). Complement in Monoclonal Antibody Therapy of Cancer. Immunol Res..

[265] Wang S.Y., Weiner G. (2008). Complement and cellular cytotoxicity in antibody therapy of cancer. Expert Opin. Biol. Ther..

[266] Van Spriel A.B., Leusen J.H.W., Van Egmond M., Dijkman H.B.P.M., Assmann K.J.M., Mayadas T.N., Van de Winkel J.G.J. (2001). Mac-1 (CD11b/CD18) is essential for Fc receptor-mediated neutrophil cytotoxicity and immunologic synapse formation. Blood.

[267] Meyer S., Leusen J.H.W., Boross P. (2014). Regulation of complement and modulation of its activity in monoclonal antibody therapy of cancer. MAbs.

[268] Reff M.E., Carner K., Chambers K.S., Chinn P.C., Leonard J.E., Raab R., Newman R.A., Hanna N., Anderson D.R. (1994). Depletion of B Cells In Vivo by a Chimeric Mouse Human Monoclonal Antibody to CD20. Blood.

[269] Harjunpää A., Junnikkala S., Meri S. (2000). Rituximab (anti-CD20) therapy of B-cell lymphomas: Direct complement killing is superior to cellular effector mechanisms. Scand. J. Immunol..

[270] Gennari R., Menard S., Fagnoni F., Ponchio L., Scelsi M., Tagliabue E., Castiglioni F., Villani L., Magalotti C., Gibelli N. (2004). Pilot study of the mechanism of action of preoperative trastuzumab in patients with primary operable breast tumors overexpressing HER2. Clin. Cancer Res..

[271] Hargadon K.M., Johnson C.E., Williams C.J. (2018). Immune checkpoint blockade therapy for cancer: An overview of FDA-approved immune checkpoint inhibitors. Int. Immunopharmacol..

[272] Agata Y., Kawasaki A., Nishimura H., Ishida Y., Tsubata T., Yagita H., Honjo T. (1996). Expression of the PD-1 antigen on the surface of stimulated mouse T and B lymphocytes. Int. Immunol..

[273] Ishida Y., Agata Y., Shibahara K., Honjo T. (1992). Induced expression of PD-1, a novel member of the immunoglobulin gene superfamily, upon programmed cell death. EMBO J..

[274] Freeman G.J., Long A.J., Iwai Y., Bourque K., Chernova T., Nishimura H., Fitz L.J., Malenkovich N., Okazaki T., Byrne M.C. (2000). Engagement of the PD-1 Immunoinhibitory Receptor by a Novel B7 Family Member Leads to Negative Regulation of Lymphocyte Activation. J. Exp. Med..

[275] Keir M.E., Liang S.C., Guleria I., Latchman Y.E., Qipo A., Albacker L.A., Koulmanda M., Freeman G.J., Sayegh M.H., Sharpe A.H. (2006). Tissue expression of PD-L1 mediates peripheral T cell tolerance. J. Exp. Med..

[276] Fife B.T., Pauken K.E., Eagar T.N., Obu T., Wu J., Tang Q., Azuma M., Krummel M.F., Bluestone J.A. (2009). Interactions between PD-1 and PD-L1 promote tolerance by blocking the TCR-induced stop signal. Nat. Immunol..

[277] Iwai Y., Ishida M., Tanaka Y., Okazaki T., Honjo T., Minato N. (2002). Involvement of PD-L1 on tumor cells in the escape from host immune system and tumor immunotherapy by PD-L1 blockade. Proc. Natl. Acad. Sci. USA.

[278] Topalian S.L., Hodi F.S., Brahmer J.R., Gettinger S.N., Smith D.C., McDermott D.F., Powderly J.D., Carvajal R.D., Sosman J.A., Atkins M.B. (2012). Safety, Activity and Immune Correlates of Anti-PD-1 Antibody in Cancer. N. Engl. J. Med..

[279] Hamid O., Robert C., Daud A., Hodi F.S., Hwu W.J., Kefford R., Wolchok J.D., Hersey P., Joseph R.W., Weber J.S. (2013). Safety and tumor responses with lambrolizumab (anti-PD-1) in melanoma. N. Engl. J. Med..

[280] Migden M.R., Rischin D., Schmults C.D., Guminski A., Hauschild A., Lewis K.D., Chung C.H., Hernandez-Aya L., Lim A.M., Chang A.L.S. (2018). PD-1 blockade with cemiplimab in advanced cutaneous squamous-cell carcinoma. N. Engl. J. Med..

[281] Vaddepally R.K., Kharel P., Pandey R., Garje R., Chandra A.B. (2020). Review of indications of FDA-approved immune checkpoint inhibitors per NCCN guidelines with the level of evidence. Cancers.

[282] Nowicki T.S., Hu-Lieskovan S., Ribas A. (2018). Mechanisms of Resistance to PD-1 and PD-L1 blockade. Cancer J..

[283] Sharma P., Hu-Lieskovan S., Wargo J.A., Ribas A. (2017). Primary, Adaptive, and Acquired Resistance to Cancer Immunotherapy. Cell.

[284] Melero I., Berman D.M., Aznar M.A., Korman A.J., Gracia J.L.P., Haanen J. (2015). Evolving synergistic combinations of targeted immunotherapies to combat cancer. Nat. Rev. Cancer.

[285] Weber J.S., Sznol M., Sullivan R.J., Blackmon S., Boland G., Kluger H.M., Halaban R., Bacchiocchi A., Ascierto P.A., Capone M. (2018). A Serum Protein Signature Associated with Outcome after Anti–PD-1 Therapy in Metastatic Melanoma. Cancer Immunol. Res..

[286] An L.L., Gorman J.V., Stephens G., Swerdlow B., Warrener P., Bonnell J., Mustelin T., Fung M., Kolbeck R. (2016). Complement C5a induces PD-L1 expression and acts in synergy with LPS through Erk1/2 and JNK signaling pathways. Sci. Rep..

[287] Pio R., Ajona D., Lambris J.D. (2013). Complement inhibition: A promising concept for cancer treatment. Semin. Immunol..

[288] Kolev M., Markiewski M.M. (2018). Targeting complement-mediated immunoregulation for cancer immunotherapy. Semin. Immunol..

[289] Naing A., Papadopoulos K.P., Autio K.A., Ott P.A., Patel M.R., Wong D.J., Falchook G.S., Pant S., Whiteside M., Rasco D.R. (2016). Safety, antitumor activity, and immune activation of pegylated recombinant human interleukin-10 (AM0010) in patients with advanced solid tumors. J. Clin. Oncol..

[290] Naing A., Infante J.R., Papadopoulos K.P., Chan I.H., Shen C., Ratti N.P., Rojo B., Autio K.A., Wong D.J., Patel M.R. (2018). PEGylated IL-10 (Pegilodecakin) Induces Systemic Immune Activation, CD8+ T Cell Invigoration and Polyclonal T Cell Expansion in Cancer Patients. Cancer Cell.

[291] Zha H., Han X., Zhu Y., Yang F., Li Y., Li Q., Guo B., Zhu B. (2017). Blocking C5aR signaling promotes the anti-tumor efficacy of PD-1/PD-L1 blockade. Oncoimmunology.

[292] Zha H., Wang X., Zhu Y., Chen D., Han X., Yang F., Gao J., Hu C., Shu C., Feng Y. (2019). Intracellular activation of complement C3 leads to PD-L1 antibody treatment resistance by modulating tumor-associated macrophages. Cancer Immunol. Res..

[293] Massard C., Cassier P., Bendell J.C., Marie D.B., Blery M., Morehouse C., Ascierto M., Zerbib R., Mitry E., Tolcher A.W. (2019). Preliminary results of STELLAR-001, a dose escalation phase I study of the anti-C5aR, IPH5401, in combination with durvalumab in advanced solid tumours. Ann. Oncol..

[294] Wang Y., Zhang H., He Y.W. (2019). The Complement Receptors C3aR and C5aR Are a New Class of Immune Checkpoint Receptor in Cancer Immunotherapy. Front. Immunol..

[295] Peng W., McKenzie J.A., Hwu P. (2016). Complementing T-cell function: An inhibitory role of the complement system in T-cell-mediated antitumor immunity. Cancer Discov..

[296] Kwan W.H., van der Touw W., Paz-Artal E., Li M.O., Heeger P.S. (2013). Signaling through C5a receptor and C3a receptor diminishes function of murine natural regulatory T cells. J. Exp. Med..

[297] Mamidi S., Höne S., Teufel C., Sellner L., Zenz T., Kirschfink M. (2015). Neutralization of membrane complement regulators improves complement-dependent effector functions of therapeutic anticancer antibodies targeting leukemic cells. Oncoimmunology.

[298] Hu W., Ge X., You T., Xu T., Zhang J., Wu G., Peng Z., Chorev M., Aktas B.H., Halperin J.A. (2011). Human CD59 inhibitor sensitizes rituximab-resistant lymphoma cells to complement-mediated cytolysis. Cancer Res..

[299] Zhao W.-P., Zhu B., Duan Y.-Z., Chen Z.-T. (2009). Neutralization of complement regulatory proteins CD55 and CD59 augments therapeutic effect of herceptin against lung carcinoma cells. Oncol. Rep..

[300] Wang Y., Yang Y.J., Wang Z., Liao J., Liu M., Zhong X.-R., Zheng H., Wang Y.-P. (2017). CD55 and CD59 expression protects HER2-overexpressing breast cancer cells from trastuzumab-induced complement-dependent cytotoxicity. Oncol. Lett..

[301] Kolev M., Towner L., Donev R. (2011). Complement in cancer and cancer immunotherapy. Arch. Immunol. Ther. Exp. (Warsz)..

[302] Bowen W.S., Svrivastava A.K., Batra L., Barsoumian H., Shirwan H. (2018). Current challenges for cancer vaccine adjuvant development. Expert Rev. Vaccines.

[303] Morgan E.L., Morgan B.N., Stein E.A., Vitrs E.L., Thoman M.L., Sanderson S.D., Phillips J.A. (2010). Enhancement of in vivo and in vitro immune functions by a conformationally biased, response-selective agonist of human C5a: Implications for a novel adjuvant in vaccine design. Vaccine.

[304] Hegde G.V., Meyers-Clark E., Joshi S.S., Sanderson S.D. (2008). A conformationally-biased, response-selective agonist of C5a acts as a molecular adjuvant by modulating antigen processing and presentation activities of human dendritic cells. Int. Immunopharmacol..

[305] Hung C.Y., Hurtgen B.J., Bellecourt M., Sanderson S.D., Morgan E.L., Cole G.T. (2012). An agonist of human complement fragment C5a enhances vaccine immunity against Coccidioides infection. Vaccine.

[306] Floreani A.A., Gunselman S.J., Heires A.J., Hauke R.J., Tarantolo S., Jackson J.D. (2007). Novel C5a agonist-based dendritic cell vaccine in a murine model of melanoma. Cell Cycle.

[307] Kollessery G., Nordgren T.M., Mittal A.K., Joshi S.S., Sanderson S.D. (2011). Tumor-specific peptide-based vaccines containing the conformationally biased, response-selective C5a agonists EP54 and EP67 protect against aggressive large B cell lymphoma in a syngeneic murine model. Vaccine.

[308] Platt J.L., Silva I., Balin S.J., Lefferts A.R., Farkash E., Ross T.M., Carroll M.C., Cascalho M. (2017). C3d regulates immune checkpoint blockade and enhances antitumor immunity. JCI insight.

[309] Xu G.L., Zhang K.Q., Guo B., Zhao T.-T., Yang F., Jiang M., Wang Q.H., Shang Y.H., Wu Y.Z. (2010). Induction of protective and therapeutic antitumor immunity by a DNA vaccine with C3d as a molecular adjuvant. Vaccine.

